# Chronic circadian rhythm disorder induces heart failure with preserved ejection fraction-like phenotype through the Clock-sGC-cGMP-PKG1 signaling pathway

**DOI:** 10.1038/s41598-024-61710-2

**Published:** 2024-05-11

**Authors:** Yiyang Che, Yuuki Shimizu, Takumi Hayashi, Junya Suzuki, Zhongyue Pu, Kazuhito Tsuzuki, Shingo Narita, Rei Shibata, Toyoaki Murohara

**Affiliations:** 1 Department of Cardiology, Nagoya University Graduate School of Medicine, 65 Tsurumai, Showa-ku, Nagoya, 466-8550 Japan; 2 Department of Advanced Cardiovascular Therapeutics, Nagoya University Graduate School of Medicine, Nagoya, 466-8550 Japan

**Keywords:** Jetlag, HFpEF, Clock, Soluble guanylate cyclase, Cyclic GMP, Riociguat, Cardiology, Molecular medicine

## Abstract

Emerging evidence has documented that circadian rhythm disorders could be related to cardiovascular diseases. However, there is limited knowledge on the direct adverse effects of circadian misalignment on the heart. This study aimed to investigate the effect of chronic circadian rhythm disorder on heart homeostasis in a mouse model of consistent jetlag. The jetlag model was induced in mice by a serial 8-h phase advance of the light cycle using a light-controlled isolation box every 4 days for up to 3 months. Herein, we demonstrated for the first time that chronic circadian rhythm disorder established in the mouse jetlag model could lead to HFpEF-like phenotype such as cardiac hypertrophy, cardiac fibrosis, and cardiac diastolic dysfunction, following the attenuation of the Clock-sGC-cGMP-PKG1 signaling. In addition, clock gene knock down in cardiomyocytes induced hypertrophy via decreased sGC-cGMP-PKG signaling pathway. Furthermore, treatment with an sGC-activator riociguat directly attenuated the adverse effects of jetlag model-induced cardiac hypertrophy, cardiac fibrosis, and cardiac diastolic dysfunction. Our data suggest that circadian rhythm disruption could induce HFpEF-like phenotype through downregulation of the clock-sGC-cGMP-PKG1 signaling pathway. sGC could be one of the molecular targets against circadian rhythm disorder-related heart disease.

## Introduction

Living organisms on the Earth have an ecological rhythm composed of a circadian clock in the body to adapt to the 24-h cycle owing to the Earth's rotation^1^. The circadian clock is genetically controlled, and mutations in clock genes can alter rhythmic behavior in animals^2^. In the main loop, which forms the basis of the oscillation control mechanism of the clock genes, the positive elements that drive the circadian cycle are the transcription factors BMAL1 and CLOCK^2^. Conversely, the transcription factor CLOCK–BMAL1 induces the expression of its own negative regulators: the period (PER) and cryptochrome (CRY) proteins. PER and CRY repress the expression of CLOCK-BMAL1 by inhibiting its transcriptional activity. Once PER and CRY levels are sufficiently decreased, a new cycle of transcription by CLOCK–BMAL1 can begin. As such, an intricate balance in the protein expression of clock genes produces a 24-h cycle^2^. 

Jetlag, a serious social issue can produce many symptoms, such as nighttime insomnia and loss of appetite^3^. It also has numerous adverse effects on health outcomes related to shift work including an increased risk of sleep alteration and fatigue, cardiovascular diseases, and obesity^4^. In addition, disruption of circadian rhythms modulates peripheral clock gene expression in the body and has adverse effects on reparative angiogenesis in a murine model^5^.

However, whether circadian misalignment has a direct adverse effect on the physiological heart are not fully understood. Moreover, the precise mechanism of circadian-controlled molecules involved in it is still unclear. Accordingly, this study was aimed to investigate the effect of chronic circadian rhythm disorders on heart homeostasis and a potential therapeutic option against it.

## Results

### Chronic circadian disorder induced cardiac hypertrophy, cardiac fibrosis, and diastolic dysfunction in WT mice

**Figure 1:**
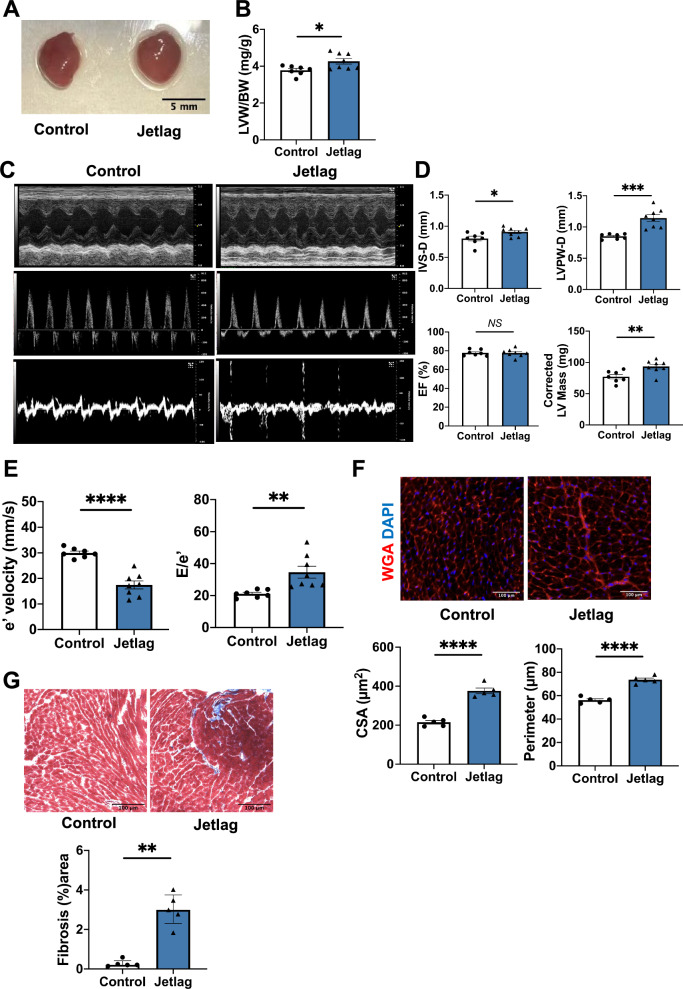
Jetlag mice exhibit cardiac hypertrophy, fibrosis, and impaired cardiac diastolic function at 3 months of the study. (**A**) Representative images of hearts after 3-month jetlag condition. The Black scale bar represents 5 mm. (**B**) Mouse Left Ventricular (LV) weight normalized to body weight in each group (control, n = 7; jetlag, n = 8). (**C**) Representative echocardiographic images of M-mode, transmitral flow velocity, and diastolic tissue velocity at 3-month induced jetlag condition. (**D**) Increased interventricular septum thickness (IVS-D) and LV posterior wall thickness at end-diastole (LVPW-d) with preserved ejection fraction (EF) and increased corrected LV mass in the jetlag group. (control, n = 7; jetlag, n = 8) (**E**) Reduced mitral valve e′ velocity and increased E–e′ ratio under 3 months jetlag condition. (Control, n = 7; jetlag, n = 8) (**F**) Representative cardiomyocyte images stained by wheat-germ-agglutinin staining (WGA) of mice hearts from control and jetlag model at 3 months. The white scale bar represents 100 µm (×20 magnification). And quantification of myocyte CSA (cross-sectional area) and perimeter. (n = 5 per group) (**G**) Representative histopathology images stained by Masson’s Trichrome of mice heart at papillary muscle level under 3-month jetlag condition. The Black scale bar represents 100 µm (×20 magnification). Quantitate analysis of the fibrosis area in control and jetlag mice hearts at 3 months. (n = 5 per group) Data are shown as mean ± SEM and analyzed by unpaired Student t-test (**B**, **D**, **E**, and **F**) or median with IQR and analyzed by Mann–Whitney test (**G**). NS indicates no significant difference. *p < 0.05, **p < 0.01, ***p < 0.001, and ****p < 0.0001 for indicated comparisons.

To determine whether chronic circadian disorder could lead to the development of cardiovascular abnormalities in mice, we constructed a mouse jetlag model by an 8-h advance once every 4 days for up to 3 months. At 3 months, the heart of the jetlag group showed cardiac enlargement compared with that of the control group (Fig. 1A). The left ventricular weight normalized by body weight showed a significant increase in the jetlag group (Fig. 1B). Also, the jetlag condition did not change the food and water consumption of mice (Figures S2A and S2B). Next, we assessed the cardiac function using echocardiography. Changes in M-mode, transmitral flow velocity, and diastolic tissue velocity in the jetlag group were noted (Fig. 1C). There was significantly increased interventricular septum thickness (IVS-D), LV posterior wall thickness at end-diastole (LVPW-d), and corrected LV mass in the systolic function of jetlag mice with preserved ejection fraction (Fig. 1D). In addition, jetlag mice had increased E to e′ ratio and decreased mitral valve e′ velocity of diastolic function (Fig. 1E). These parameters indicate that chronic jetlag could lead to impaired cardiac diastolic function with a preserved ejection fraction. Furthermore, histological analysis revealed obvious cardiac hypertrophy and fibrosis in the heart of the jetlag mice (Fig. 1F,G). Also, the qPCR results in 1-month jetlag model also showed increased cardiac hypertrophy related gene expression, supporting the above data (Figure S2E, S2F and S2G). In addition, capillary rarefaction was observed in 3-months Jetlag group (Figure S2I).

### Differential gene expression induced following 1-month constant jetlag condition

**Figure 2:**
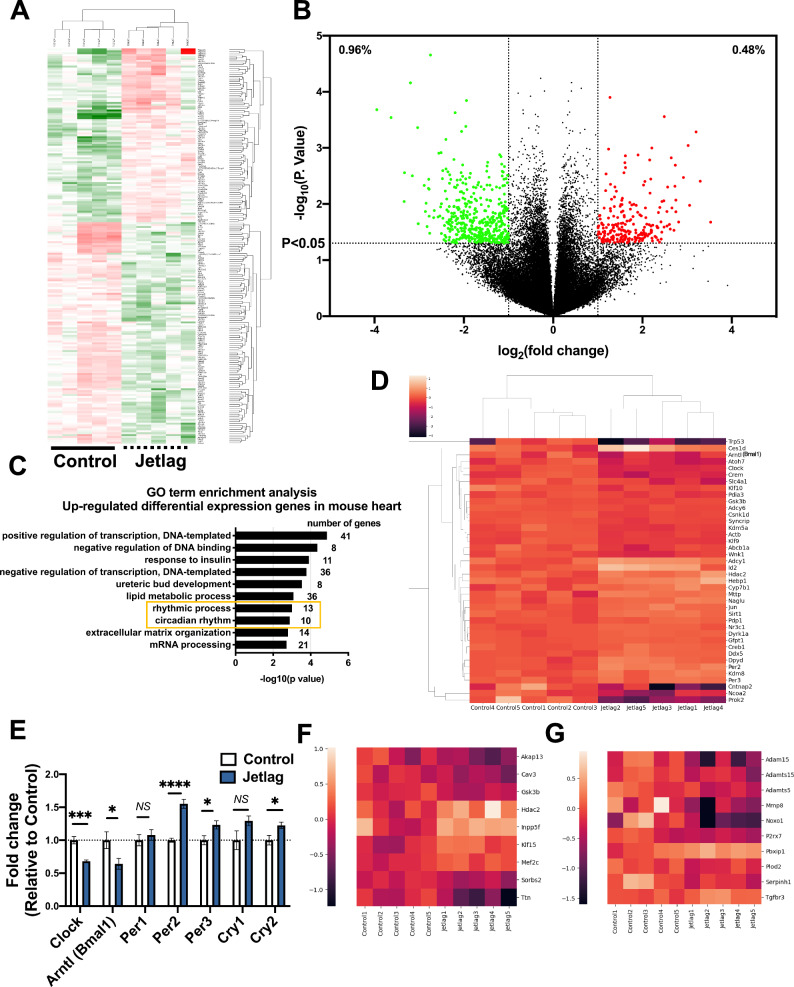
Bioinformatics analysis of mouse heart tissue following circadian disorder at 1 month (light phase). (**A**) Heatmap of significantly changed genes between control and jetlag group. Red represents increased abundance. Green represents decreased abundance (n = 5 per group). (**B**) Volcano plot displaying genes expression in mouse heart between control (n = 5) and jetlag (n = 5) group at 1 month. The two vertical lines are the log2fold change boundaries and the horizontal line is the statistical significance boundary (*p* value < 0.05). There were 218 up-regulated genes (right, red) and 431 down-regulated genes (left, green). (**C**) The top 10 GO terms in the biological process are presented in the enrichment analyses of all significantly upregulated genes in jetlag mouse hearts. (**D**) Clustermap of circadian rhythm-related genes from differential expressed genes between control and jetlag group. Normalized data from each group were used (n = 5 per group). (**E**) Circadian rhythm-related gene expression levels between the control and jetlag groups were shown by raw data (n = 5 per group). (**F**) Heatmap of cardiac hypertrophy-related genes from differential expressed genes between control and jetlag group. Normalized data from each group were used (n = 5 per group). (**G**) Heatmap of collagen metabolic process and extracellular matrix disassembly related genes from differential expressed genes between control and jetlag group. Normalized data from each group were used (n = 5 per group). Figure E are shown as mean ± SEM and analyzed by unpaired Student t-test. *p < 0.05, ***p < 0.001, and ****p < 0.0001 for indicated comparisons.

To comprehensively assess the changes in gene expression induced by chronic jetlag conditions, we performed microarray analysis on mouse heart tissue collected at light phase after exposure to a 1-month of constant jetlag condition. Microarray analysis revealed there are multiple gene expression changes in the 1-month jetlag model (Fig. 2A). We identified 2065 differentially expressed genes (DEGs) between the control and jetlag groups with *p* value < 0.05 and 1.2 < fold change < 0.8 (Fig. 2B). A total of 825 up-regulated genes were identified and subjected to Gene Ontology (GO) term enrichment analysis. Among the top 10 GO terms in biological process, “rhythmic process” and “circadian rhythm” were significantly changed in the 1-month jetlag condition (Fig. 2C). Also, the TOP5 GO Pathways showed the impact DNA regulation and repair system in Jetlag model, which is consistent with the results other paper showed^6–8^. Cluster maps from DEGs selected by the GO term circadian rhythm (GO: 0007623) also showed that multiple circadian rhythm-related genes were altered in the 1-month jetlag group (Fig. 2D). Detailed clock gene expression levels are also shown using the raw data (Fig. 2E). Given the cardiac hypertrophy and fibrosis in the jetlag group heart at 3-months, as revealed by histological analysis, we also checked the expression of cardiac hypertrophy and fibrosis-related genes (GO: 0003300; 0032963; 0022617) in DEGs. The heatmap showed significant changes in cardiac hypertrophy related gene expression such as Ttn and fibrosis related gene expression, supporting the above data (Fig. 2F,G).

### Circadian disorder at 1 month disrupted the sGC-cGMP-PKG signaling

**Figure 3:**
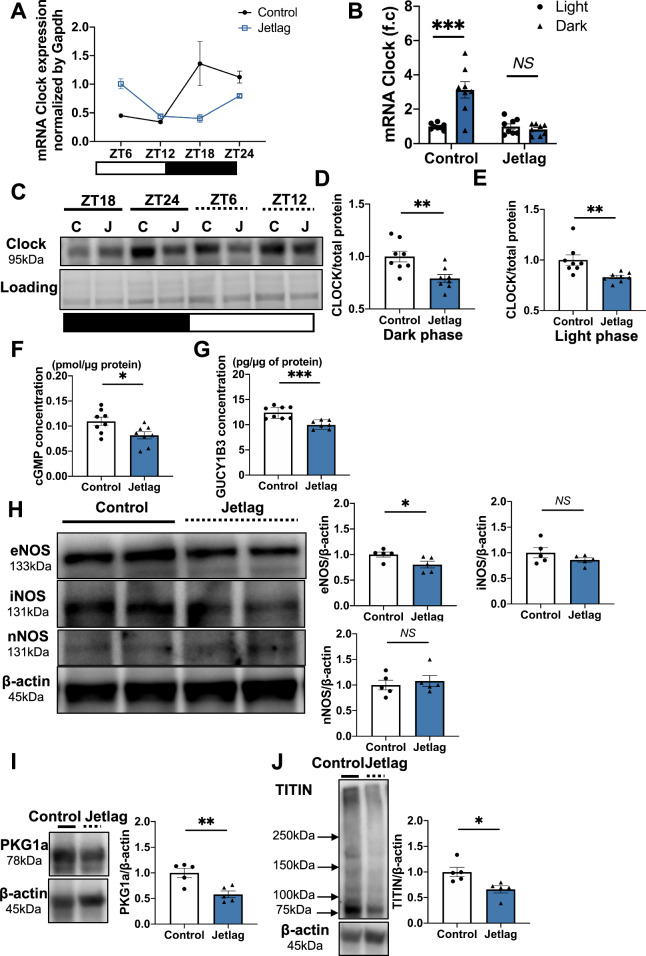
CLOCK protein expression and NO-sGC-cGMP signaling pathway at 1 month of the experiment. (**A**) Diurnal rhythms in *Clock* mRNA expression by qRT-PCR in 1-month control and jetlag hearts. (n = 4 hearts/time point/group) (**B**) Average photoperiod quantification of *Clock* mRNA expression in 1-month control and jetlag hearts. ZT6, and ZT12 were identified as light phase. ZT18, and ZT24 were identified as dark phase (n = 8 per group). (**C**) Representative western blots of CLOCK and total protein loading in 1-month control and jetlag heart at different time point. Average photoperiod quantification of CLOCK protein expression at (**D**) dark phase and (**E**) light phase in 1-month control and jetlag hearts. ZT18, and ZT24 were identified as dark phase. ZT6, and ZT12 were identified as light phase (n = 8 per group). (**F**) cGMP (n = 8 per group) and (**G**) soluble guanylate cyclase (sGC) (control, n = 8; jetlag, n = 7) concentration normalized to protein concentration in mouse left ventricular are shown in the control and jetlag group at light phase of 1 month of the study. (**H**) Representative western blots and quantification of eNOS, iNOS, nNOS, and beta-actin from 1-month control and jetlag heart at light phase (n = 5 per group). (**I**) Representative western blots and quantification of PGC1a and beta-actin from 1-month control and jetlag heart at light phase (n = 5 per group). (**J**) Representative western blots and quantification of TITIN and beta-actin from 1-month control and jetlag heart at light phase (n = 5 per group). Data are shown as mean ± SEM and analyzed by unpaired Student t-test (**B**, **D**, **E**, **F**, **H**, **I** and **J**) or median with IQR and analyzed by Mann–Whitney test (G). *p < 0.05, **p < 0.01, ***p < 0.001, and ****p < 0.0001 for indicated comparisons.

Since we identified the significant decrease of Clock gene expression in jetlag group from microarray data and its core role in circadian mechanism, we try to further identify the rhythmic expression of Clock gene in jetlag group. There is also a significant relationship between the NO-cGMP-PKG signaling pathway and HFpEF. Therefore, we also investigated whether cardiac diastolic dysfunction with preserved ejection fraction and cardiac hypertrophy and fibrosis in the jetlag group developed through modulation of the NO-cGMP-PKG pathway. qRT-PCR analysis revealed rhythmic change at different time point of Clock mRNA in 1 month heart (Fig. 3A). To further confirm the rhythmic expression, we plot Clock mRNA values over the light or dark phase in both control and jetlag group. And results showed clock expression in jetlag group exhibited a loss of rhythm (Fig. 3B). We also found that in both light or dark phase, the CLOCK protein expression was decreased in jetlag group (Fig. 3C–E). In addition, eNOS expression at the protein level decreased in the jetlag group after 1 month at light phase (Fig. 3H). Moreover, cGMP and soluble guanylate cyclase (sGC) concentrations at light phase in the left ventricle were downregulated in 1-month jetlag hearts (Fig. 3F,G). Furthermore, we identified that PKG1a and TITIN expression at the protein level also decreased in the jetlag group at light phase of 1 month (Fig. 3I,J).

### Clock gene knock-down in H9c2 cardiomyocytes developed hypertrophy and interrupted the sGC-cGMP-PKG signaling.

**Figure 4:**
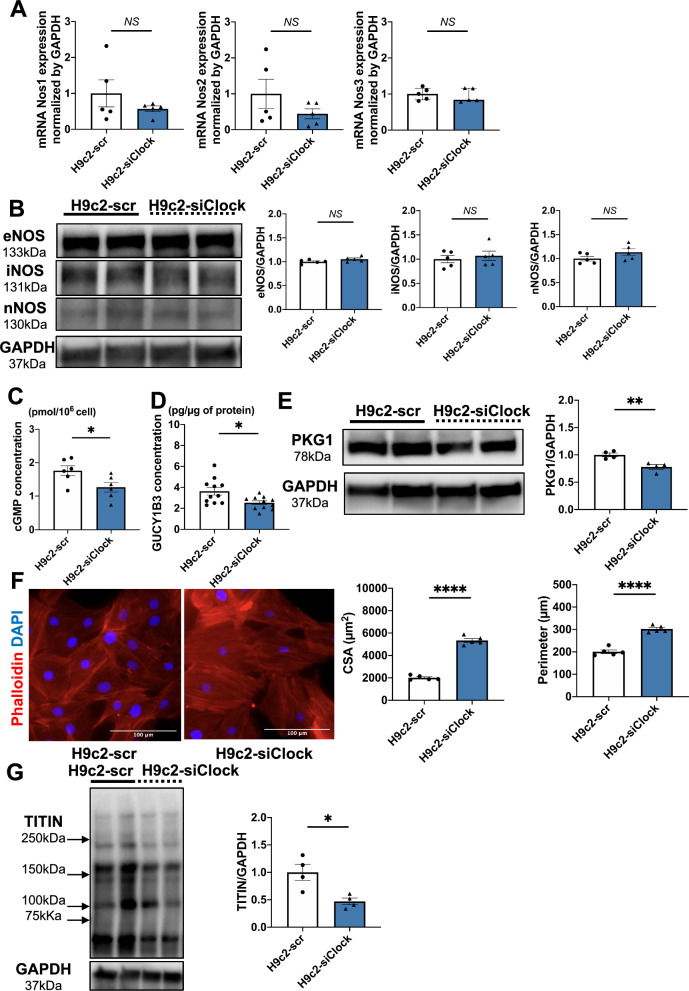
NO-cGMP-PKG signaling pathway, cell area, and TITIN protein expression in Clock knocked-down H9c2 cardiomyocytes. (**A**) *Nos1*, *Nos2*, and *Nos3* mRNA expression by qRT-PCR in H9c2-scr and Clock-KD H9c2 cardiomyocytes. (n = 5 per group) (**B**) Representative western blots and quantification of eNOS, iNOS, nNOS, and GAPDH in H9c2-scr and Clock-KD H9c2 cardiomyocytes. (n = 5 per group) (**C**) cGMP (n = 6 per group) and (**D**) soluble guanylate cyclase (sGC) (n = 11 per group) concentration in H9c2-scr and Clock-KD H9c2 cardiomyocytes. (**E**) Representative western blots and quantification of PKG1 and GAPDH in H9c2-scr and Clock-KD H9c2 cardiomyocytes (n = 4 per group). (**F**) Representative phalloidin staining (red) of actin filaments in H9c2-scr and Clock-KD H9c2 cardiomyocytes. Cell nuclei were stained by DAPI (blue). And H9c2 cardiomyocyte size was quantified by cell surface area and cell perimeter (n = 5 per group). (**G**) Representative western blots and quantification of TITIN and GAPDH in H9c2-scr and Clock-KD H9c2 cardiomyocytes (n = 4 per group). Data are shown as mean ± SEM and analyzed by unpaired Student t-test (**A** [*Nos1* and *Nos2*], **B**, **C**, **D**, **E**, **F** and **G**) or median with IQR and analyzed by Mann–Whitney test (A [*Nos3*]). *p < 0.05, **p < 0.01, and ****p < 0.0001 for indicated comparisons.

To further investigate relationship between circadian rhythm-related genes and the NO-cGMP-PKG signaling pathway, we next focused on the clock gene. After the knockdown of the clock gene in H9c2, the mRNA and protein levels of Clock is significantly decreased in H9c2-siClock group (Figure S3A). And the mRNA and protein levels of *Nos1*, *Nos2*, and *Nos3* were not significantly changed (Fig. 4A,B). However, cGMP and soluble guanylate cyclase (sGC) concentration in Clock knock down H9c2 cells decreased (Fig. 4C,D). The PKG1 protein level was also significantly downregulated in Clock knock down H9c2 cardiomyocytes (Fig. 4E). Furthermore, Clock knock down H9c2 cardiomyocytes were larger in size than WT H9c2 cardiomyocytes (Fig. 4F). And in H9c2-siClock group, the cardiac hypertrophy related genes expression is upregulated (Figure S3B). Clock knock down H9c2 cardiomyocytes also exhibited downregulation of TITIN expression at the protein level (Fig. 4G).

### Riociguat treatment improved cardiac hypertrophy, cardiac fibrosis, and diastolic dysfunction induced by constant jetlag conditions.

**Figure 5:**
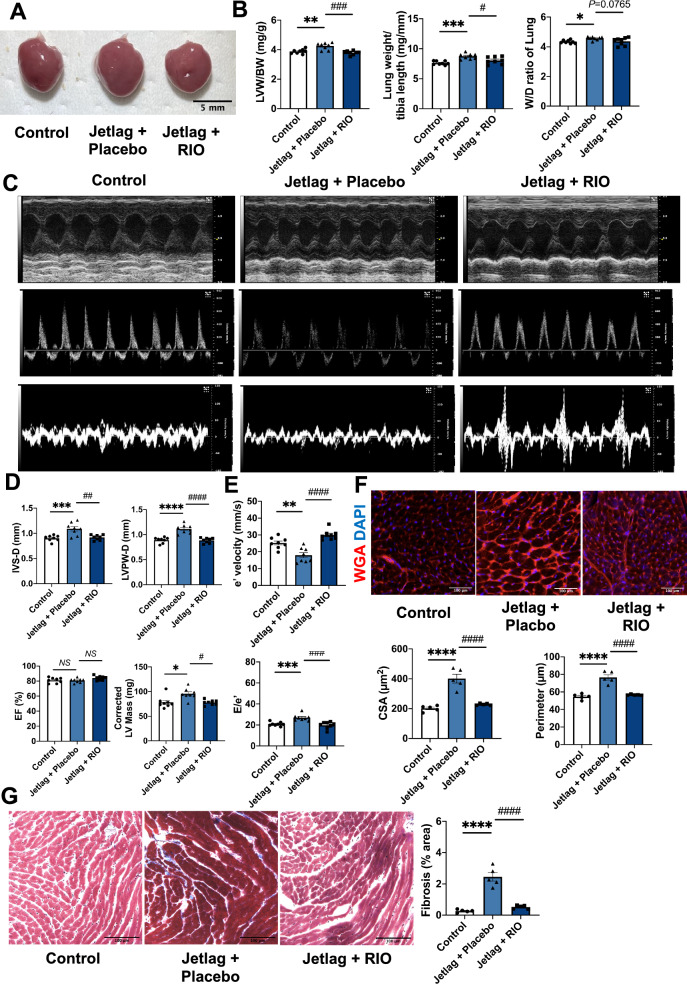
Riociguat treatment alleviated adverse myocardial remodeling in constant jetlag conditions. (**A**) Representative images of hearts after a 3-month jetlag condition or 3-month treatment of Riociguat. The Black scale bar represents 5 mm. (**B**) Mouse Left Ventricular (LV) weight normalized to body weight, lung weight normalized to tibia length, and lung W/D ratio in each group (n = 8 per group). (**C**) Representative echocardiographic images of M-mode, transmitral flow velocity, and diastolic tissue velocity at 3 months of the study. (**D**) Interventricular septum thickness (IVS-D), LV posterior wall thickness at end-diastole (LVPW-d), ejection fraction (EF) and corrected LV mass in each group. (n = 8 per group) (**E**) Mitral valve e′ velocity and E–e′ ratio in each group. (n = 8 per group) (**F**) Representative cardiomyocyte images stained by wheat-germ-agglutinin staining (WGA) of mice hearts at 3 months. The white scale bar represents 100 µm (×20 magnification). And quantification of myocyte CSA (cross-sectional area) and perimeter. (n = 5 per group) (**G**) Representative histopathology images stained by Masson’s Trichrome of mice heart at papillary muscle level and the cutting direction is the short axis under 3-month jetlag condition or jetlag condition with the treatment of Riociguat. The Black scale bar represents 100 µm (×20 magnification). Quantitate analysis of the fibrosis area in each group. (n = 5 per group) Data are shown as mean ± SEM and analyzed by one-way analysis of variance (ANOVA) with Tukey’s multiple comparisons test (**B**, **D** [IVS-D, LVPW-D, and EF], **E**, **F**, and **G**) or analyzed by Kruskall–Wallis test with Dunn multiple comparisons test (**D** [Corrected LV Mass]). *p < 0.05, **p < 0.01, ***p < 0.001, ****p < 0.0001 significant vs. control; ^#^*p* < 0.05, ^##^*p* < 0.01, ^###^*p* < 0.001, ^####^*p* < 0.0001 significant versus Jetlag.

Finally, we treated mice with a sGC stimulator riociguat for 3 months to test whether cardiac diastolic dysfunction with preserved ejection fraction in the jetlag group was prevented or not. At 3 months of the study, the heart of the treatment group was smaller than that of the non-treated jetlag group (Fig. 5A). And left ventricular weight normalized by body weight and lung weight normalized by tibia length in the treatment group showed a significant decrease compared with those in the non-treated jetlag group (Fig. 5B). The lung wet-to-dry ratio in the jetlag group was higher than that in the control group (Fig. 5B). Echocardiography analysis showed that the increased interventricular septum thickness (IVS-D), LV posterior wall thickness at end-diastole (LVPW-d), and corrected LV mass in the jetlag mice was significantly relieved in the treatment group (Fig. 5C,D). In addition, cardiac diastolic function induced by constant jetlag conditions was ameliorated by riociguat (Fig. 5E). Histological analysis similarly showed attenuated hypertrophy and fibrosis in the Riociguat treatment group (Fig. 5F,G).

## Discussion

The major findings of the present study are as follows: (1) Chronic circadian rhythm disorder established in the mouse jetlag model could lead to HFpEF-like phenotype such as cardiac hypertrophy, cardiac fibrosis, and cardiac diastolic dysfunction with preserved ejection fraction. (2) Biological processes, such as rhythmic process, circadian rhythm, cardiac hypertrophy, and fibrosis-related genes in the heart were significantly altered in the jetlag model. (3) Circadian rhythm disruption decreases the Clock-sGC-cGMP-PKG1 signaling in the heart. (4) Clock gene knock down in cardiomyocytes induced hypertrophy via decreased sGC-cGMP-PKG1 signaling pathway. (5) Treatment with an sGC-activator riociguat attenuated directly the adverse effects of jetlag model-induced cardiac hypertrophy, cardiac fibrosis, and cardiac diastolic dysfunction.

Circadian rhythms, which are regulated by circadian clocks, are biological rhythms that enable mammals to adjust to daily oscillations caused by light–dark cycles by coordinating internal biological activities with environmental changes^9^. In circadian clocks, the central clock coordinated with clock genes that exist in peripheral tissues maintains a normal 24-h cycle and is responsible for the diurnal rhythm. Circadian disruption caused by jetlag, shift work, or exposure to artificial light at night could have wide-ranging adverse effects on both physical and mental health, and induce poor health outcomes^10^. Circadian rhythms also play a significant role in the regulation of cardiovascular physiology and disease. In the cardiovascular system, different types of cells have peripheral clocks that control various physiological and pathological processes, including endothelial function, blood pressure, heart rate, arterial stiffness^11^, hypertension^12^, myocardial infarction^13^, coronary heart disease^14–16^, heart failure^17–19^, stroke^20^, and atherosclerosis^21^. In animal models, circadian disruption were reported to lead to cardiovascular disease^22,23^. Therefore, using the mouse jetlag model by shifting the light period to mimic clinical conditions, such as shift work, this study aimed to investigate the effect of chronic circadian disruption on heart homeostasis.

Nearly half of all patients with heart failure (HF) worldwide suffer from HF with preserved ejection fraction (HFpEF), a major contributor of morbidity and mortality, and its incidence is increasing alarmingly. Patients with HFpEF develop classic symptoms of HF, and their pathophysiology involves a multi-organ syndrome in which many components combine to cause symptoms and outcomes^24^. Clinical epidemiological data reported that sleep disorders could be a risk factor for HFpEF, regardless of the lack of a mechanism^25^. A mouse model of circadian disorders exhibited indirect cardiac hypertrophy, cardiac stiffness, and/or cardiac diastolic function^26,27^. To our knowledge, this is the first study to demonstrate that mice under chronic jetlag conditions develop multiple levels of cardiovascular diseases, including cardiac hypertrophy and fibrosis, resulting in diastolic dysfunction via at least in part of the modulation of clock genes in the heart. As few effective pharmacotherapies or treatments are available for HFpEF, elucidating the unknown molecular mechanisms in HFpEF owing to chronic circadian rhythm disturbances is of great clinical significance.

The direct relationship between clock genes and signaling related to the onset and/or progression of HFpEF is not fully understood. Circadian rhythms are driven by molecular clocks involving core clock genes such as *BMAL1*, *CLOCK, PER1, PER2,* and *CRY1/2*. As a positive element, the transcription factor CLOCK-BMAL1 drives the circadian cycle and induces the expression of its own negative regulators, the PER and CRY proteins. Once the period and cryptochrome levels are sufficiently decreased, a new cycle of transcription by CLOCK–BMAL1 can begin. In addition to circadian disruption, clock gene depletion can lead to negative cardiac remodeling and aggravate cardiovascular diseases^28–31^. In this study, we focused on chronic jetlag condition induced by *Clock* down-regulation and its relationship with the following pathological changes in the heart. Recently, it has been reported that cardiomyocyte-specific Clock mutant (CCM) mice developed age-dependent cardiac hypertrophy and fibrosis^32^. Regarding the involved pathway between decreased *Clock* and cardiac negative remodeling, Alibhai reported *Clock* is a key regulator of the AKT signaling for increased heart weight, cardiomyocyte hypertrophy, interstitial fibrosis, and reduced left ventricular function^33^. These findings are consistent with the results of our KEGG pathway analysis of the RNA-sequencing data (Supplemental Fig. 2K).

As a secondary messenger, cyclic guanosine monophosphate (cGMP) regulates multiple cell types and organs. In addition, it is a key messenger in the regulation of physiological homeostasis. cGMP production can be stimulated by nitric oxide with sGC. The NO-sGC-cGMP signaling pathway plays a critical role in the regulation of hypertrophy, fibrosis, and angiogenesis response of the cardiovascular system^34^. Under specific pathological conditions such as circadian disruption^35^, NO production is decreased, thus inducing dysfunction of the NO-sGC-cGMP signaling pathway, thereby leading to the development of many cardiovascular diseases, such as heart failure, pulmonary hypertension, atherosclerosis, chronic kidney diseases, and hypertension^36–39^. As a multisystemic disorder with a high morbidity and mortality, cardiomyocyte hypertrophy was associated with stiffness in patients with HFpEF, with a reduction in the NO-cGMP-PKG signaling pathway^24^. Moreover, the NO-sGC-cGMP signaling pathway is suggested to play a vital role in the treatment of HFpEF in a mouse model^40^. Our results confirmed that the sGC-cGMP-PKG1 signaling was down-regulated in the mouse jetlag model. And eNOS protein expression was decreased due to the constant circadian disorder condition. Therefore, to further validate the mechanistic link between Clock gene and cGMP-signaling pathway, we performed in vitro experiments and found that Clock-KD H9c2 cardiomyocytes also exhibited the impaired sGC-cGMP-PKG signaling pathway. But results showed that none of the NOS isoforms were altered in Clock-KD H9c2 cardiomyocytes. Therefore, we assumed clock down regulation decreased sGC expression, then disrupt the cGMP signaling pathway. Natriuretic peptide (NP)-particulate GC is another intrinsic pathway in cGMP/PKG signaling. Since our results showed decreased cGMP expression in the jetlag model, we checked the expression of NPs and their receptors in the RNA-sequencing data and found that the NP-particulate GC was not modulated by chronic circadian rhythm disorder (Supplemental Fig. 2J).

Next, we try to understand the mechanism between sGC-cGMP-PKG1 signaling and cardiac diastolic dysfunction in constant jetlag condition. As a giant structural sarcomeric filament microprotein, titin has been described to be involved in cardiac diastolic dysfunction, and post-translational modification of titin is unbalanced in HFpEF patients^41,42^. In addition, cGMP-PKG1 signaling has been discovered to regulate titin as a therapeutic target to improve cardiac diastolic dysfunction^43–45^. Consequently, we investigated titin expression in constant jetlag condition. First, microarray data showed Ttn expression is significantly decreased in 1-month jetlag model (Fig. 2F). Then, the western blot results confirmed TITIN protein level is also decreased in 1-month jetlag condition. The TITIN protein level is also decreased in Clock-KD H9c2 myocytes. Taken together, we conclude the PKG1 would decreases the titin expression in constant jetlag condition.

The NO-sGC-cGMP signaling pathway is a pharmacological target of sGC stimulators and activators. According to preclinical and clinical studies, sGC agonists have a positive influence on metabolic risk factors, such as weight gain, glucose levels, and cholesterol, and the health and function of neurons. They also reduce inflammation and fibrosis. Owing to its crucial function in the cardiovascular system, the sGC-cGMP-PKG1 signaling is of therapeutic interest, especially in the context of pulmonary hypertension (PH), systemic hypertension, and HF. As a soluble guanylate cyclase stimulator, riociguat has been approved for the treatment of pulmonary arterial hypertension (PAH) and has been widely studied for the treatment of pulmonary hypertension resulting from systolic left ventricular dysfunction and HFpEF^46,47^. However, whether riociguat treatment can ameliorate circadian rhythm disruption-induced cardiac hypertrophy, fibrosis, and diastolic function remains unknown. Here, we first demonstrated that riociguat treatment could directly protect against adverse cardiac remodeling and dysfunction induced by circadian rhythm disorders. Importantly, our findings contribute insights for the development of a novel treatment to alleviate the progression of HFpEF with circadian rhythm disturbances.

There are several limitations in our current study. First, this study investigated cardiac changes, mainly in cardiomyocytes as an in vitro study, and no experiments using fibroblast cell lines were conducted in current study. We need to demonstrate the effect of a siRNA-mediated Clock knockdown on proliferation in a fibroblast cell line in future studies.

In conclusion, chronic circadian rhythm disorder could induce cardiac hypertrophy, partly by downregulating the *clock*-sGC-cGMP-PKG1 signaling pathway, leading to cardiac diastolic dysfunction. Our findings offer insights into the elucidation of the triggers and treatment of HFpEF.

## Materials and methods

Materials and Methods are available in the manuscript or Supplemental File. The data, analytic methods, and study materials employed in this study are available from the corresponding author upon reasonable request.

### Animal studies

All animal care and use procedures in this study were approved by the Animal Ethics Review Board of the Nagoya University School of Medicine. C57BL6J male mice (age 8–10 weeks) were purchased from Charles River Laboratories Japan Inc. (Kanagawa, Japan) and used as wild-type (WT) mice. The stock number of the used Charles River mice was IMSR_JAX: 000,664. Sex influences the development of cardiovascular disease^48^. As such, we only used male mice in our studies. This allowed for the evaluation of the impact of circadian rhythm disorder on heart in a well-controlled experimental system. Mice were randomly assigned to experimental groups and anesthetized with a combination of hydrochloric acid medetomidine (0.3 mg/kg), midazolam (4 mg/kg), and butorphanol tartrate (5 mg/kg) intraperitoneally before the surgical procedure. Cervical dislocation under anesthesia was used for euthanasia^49^. This study was reported in accordance with ARRIVE guidelines.

### Mouse Jetlag model

The jetlag model was induced in C57/Bl6J (wild-type) mice (age 8–10 weeks) by exposing them to an 8-h phase advance of the light period, which was adjusted using an optional timer (MELQUEST, No. LCT-8) in a light-controlled isolation box (MELQUEST, No. MBX-002) every four days for one or three months (Supplemental Fig. 1)^5^. Mice in the control group were kept under a 12-h light:12-h dark environment (light–dark condition with 9:00 [zeitgeber time 0: ZT0]-21:00 [ZT12] light phase). All the mice in this study were provided food and water ad libitum. Blood pressure was determined in conscious mice using a tail-cuff pressure analysis system (Softron). The lung wet-to-dry weight (W/D) ratio was measured in 3 months study. Generally, wet weight of the removed lung was measured after euthanasia, and the dry weight of the lung was obtained by incubating for 48 h at 60 °C^50^.

### Treatment of riociguat

For the treatment of riociguat (Sigma-Aldrich) in vivo^51,52^, Alzet mini-osmotic pumps (Model 2006, Durect Corp.) were implanted subcutaneously to deliver 10 mg/kg/day of riociguat (RIO) or placebo (Placebo) to 8- to 10-week-old mice for up to 1 month or 3 months from the same time when the jetlag group experiment started. New Alzet mini-osmotic pumps with riociguat were changed at the end of the 6-week treatment for a 3-month study. Mice were euthanized at the end of one or three months, and samples were collected for further analysis.

### Echocardiography analysis

Echocardiographic analyses of in vivo cardiac function were performed at the beginning of the study and at 1, 2, and 3 months of the study using the Vevo 1100 imaging system (FUJIFILM Visual Sonics, Inc., Toronto, ON, Canada). Mice were anesthetized with 1–3% isoflurane supplemented with 100% O2 for the duration of measurement. B-mode and M-mode images were obtained to calculate the cardiac systolic function^53^. Apical 4-chamber views were used in anesthetized mice to obtain diastolic function measurements using pulsed-wave and tissue Doppler at the level of the mitral valve. Diastolic function was evaluated by the ratio of early diastolic mitral inflow velocity (E) to early diastolic mitral annulus velocity (e′) (E/e′ ratio) at 3 months of the study.

### Microarray data processing and analysis

Sample preparation for microarray was performed at light phase of 1-month study of hearts according to the routine methods recommended by the company. The microarray platform used in this test was the Thermo Fisher Scientific Applied Biosystems TM GeneChipTM Mouse Genome 430 2.0 Array, and the microarray and following data processing were performed by Riken Genesis (Kawasaki, Japan). The scan data was digitized with Affymetrix GeneChip® Command Console software (AGCC). The numerical values output from AGCC is imported into GeneSpring GX software for data normalization and comparative analysis by Riken Genesis (Kawasaki, Japan). The *p* value is corrected by Fisher’s exact test. A volcano plot was shown by |log2fold-change|> 1 and *p* value < 0.05 using GraphPad Prism 8^54^. The criteria for differential expression genes (DEGs) were 1.2 < fold change < 0.8 and *p* value < 0.05. Under these conditions, gene ontology (GO) analysis and KEGG pathway analysis were performed using DAVID Bioinformatics Resources 6.8^54^. Cluster map and heatmaps were created using the Seaborn cluster map and heatmap package in Python. Normalized data imported from GeneSpring GX software was also analyzed by limma package of R and further conducted to GO analysis with cut-off as *p* value < 0.05 (Table S4).

### Cell culture

H9c2 cells were purchased from ATCC (Manassas, VA, USA). Cells were grown in Dulbecco’s modified Eagle’s medium (DMEM) (Sigma) supplemented with 10% fetal bovine serum and 1% penicillin/streptomycin. Cell at passage 5–9 were used for this experiment. After H9c2 cells reached 70% confluence, siRNA targeting CLOCK (10 nM, ORIGENE) or scrambled sequence (negative control, 10 nM, Ambion) was transfected into H9c2 cells using Lipofectamine RNAi Max (ThermoFisher Scientific) for 48 h, according to the manufacturer’s instructions^5^. Next, the culture medium containing the transfecting siRNA was removed, and the cells were used for the follow-up experiment.

### Isolation of RNA and quantitative real-time PCR analysis

RNA was extracted from 1 month-study Control and Jetlag hearts at different timepoint or at light phase or H9c2 cells immediately after 48 h of transfection with siRNA targeting CLOCK using the RNeasy Mini Kit (Qiagen), and equal amount of total RNA was reverse-transcribed using ReverTra Ace qPCR RT Master Mix (TOYOBO). Real-time PCR was performed using Bio-Rad real-time PCR detection system with THUNDERBIRD SYBR qPCR mix (TOYOBO) under the following conditions: 95 °C for 10 min, followed by 40 cycles at 95 °C for 15 s and 60 °C for 45 s^55^. The gene expression of each target gene was normalized to that of GAPDH^55^. The primer sequences are listed in the Supplemental Materials.

### Western blot analysis

Heart samples from each group at one month were homogenized and lysates at light phase or at different time point were prepared as previously described. H9c2 cells were homogenized immediately after 48 h of transfection with siRNA targeting CLOCK^5^. Equal amounts of protein were subjected to CriterionTGX (Tris–Glycine extended) Stain-Free PAGE gels (BioRad) and activated using a ChemiDoc MP Visualization System (BioRad). The protein was then transferred to PVDF membranes as previously described^49,56^. Those membranes were then imaged to obtain total protein loadings. The membranes were blocked and probed with the targeted primary antibodies, followed by incubation with secondary antibodies. The protein signal was detected using an ECL Prime Western Blotting System (Cytiva). Protein expression was analyzed by measuring band intensities using Image J software^49^. Average photoperiod quantification of CLOCK protein expression was normalized to total protein. The antibodies are described in the Supplemental Materials.

### Histology and immunohistochemistry

Heart samples from each group at 3 months were embedded in an OCT compound (Sakura). Frozen Sects. (5 µm in thickness) were used for histological analysis. Heart tissue fibrosis was determined by Masson’s trichrome staining, following the manufacturer’s protocol (Sigma). The heart fibrosis area was calculated as the average percentage of fibrosis of five randomly selected fields per sample using Image J analysis. The cross-sectional area (µm^2^) and cell perimeter (µm) of cardiomyocytes in heart frozen sections were analyzed by Wheat Germ Agglutinin Staining. After fixation with 4% paraformaldehyde (PFA) and washing thrice with PBS, the sections were blocked with 1% BSA at room temperature for 1 h. The sections were then incubated with Texas Red-X conjugated wheat germ agglutinin (5:1000, Thermo Fisher Scientific) at room temperature for 1 h or with primary antibody CD31 mAb (1:500, BD Pharmingen) at 4 °C overnight, followed by incubation with secondary antibody Alexa-Flour 594 conjugated anti-rat antibody (1:1000, Thermo Fisher Scientific) at room temperature for 1 h^10^. H9c2 cell surface area was analyzed by F-actin staining as previously described. After fixation with 4% PFA and permeabilization with 0.5% Triton X-100, H9c2 cells were incubated with Alexa Fluor 594 conjugated phalloidin (5:2000, Thermo Fisher Scientific) for 1 h at room temperature according to the manufacturer's instructions. Cell nuclei were stained with 4′,6-diamidino-2-phenylindole (DAPI; 1:1000, DOJINDO)^53,57^. Images were visualized using a BZ-X710 fluorescence microscope (KEYENCE, Japan) at 20 × magnification. Five pictures were randomly taken per section, and the cell surface area and perimeter were assessed using Image J software^58^. We quantified 10 cardiomyocytes/fields for H9c2 cells and 20 cardiomyocytes/fields for heart frozen sections.

### Enzyme-linked immunosorbent assay

Mouse blood serum samples were collected at light phase of 1 and 3 months of the study. Epinephrine and norepinephrine levels in the serum samples at 1 or 3 months of the study were measured using the Epinephrine/Norepinephrine ELISA Kit (Abnova) according to the manufacturer’s protocols^53^. Concentrations of Cyclic GMP in heart tissues at 1 month and H9c2 cell lysates were measured using the Cyclic GMP Complete (Enzo) according to the manufacturer’s protocols. The concentration of the Guanylate Cyclase Soluble Subunit Beta-1 in heart tissues at 1 month was determined using a Mouse Guanylate Cyclase Soluble Subunit Beta-1 (GUCY1B3) ELISA Kit (MyBioSource) according to the manufacturer’s instructions, and the concentrations of Guanylate Cyclase Soluble Subunit Beta-1 in H9c2 cell lysates were the determined using the Rat Guanylate Cyclase Soluble Subunit Beta-1 (GUCY1B3) ELISA Kit (MyBioSource) according to the manufacturer’s instructions^53^.

### Statistical analysis

Continuous parametric data were expressed as mean ± standard error of the mean (SEM), and in the case of non-parametric data, the median with interquartile range were reported. The Shapiro–Wilk normality test was used to evaluate the data distribution. For variables with normal distribution, statistical significance was evaluated using an unpaired Student’s *t*-test between the two groups. One-way analysis of variance (ANOVA) with Tukey’s multiple comparison test, was used for three or more groups. For variables with non-normal distribution, statistical significance was analyzed using the Mann–Whitney test between two groups, and the Kruskal–Wallis test with Dunn’s multiple comparisons test was used for three or more groups^58^. GraphPad Prism software (version 8.0; GraphPad Software Inc.) was used for the statistical analysis. Statistical significance was defined as *p* < 0.05^59^.

### Supplementary Information

Supplementary Information.

## Data Availability

Materials and Methods are available in the manuscript or Supplemental File. The data, analytic methods, and materials employed in this study are available from the corresponding author upon reasonable request.

## Abbreviations

DAPI: 4′,6-Diamidino-2-phenylindole
DEGs: Differentially expressed genes
ELISA: Enzyme-linked immunosorbent assay
EF: Ejection fraction
HF: Heart failure
HFpEF: Heart failure with preserved ejection fraction
KD: Knockdown
LV: Left ventricle
PBS: Phosphate-buffered saline
qPCR: Quantitative polymerase chain reaction
RIO: Riociguat
WT: Wild type
