# Supplementary material for: Chronic circadian rhythm disorder induces heart failure with preserved ejection fraction-like phenotype through the Clock-sGC-cGMP-PKG1 signaling pathway
Source: Sci Rep. 2024 May 11;14:10777. doi: 10.1038/s41598-024-61710-2 (PMC11088651; doi:10.1038/s41598-024-61710-2)
Supplement: Supplementary file 1 — Supplementary Information. [file 41598_2024_61710_MOESM1_ESM.docx]

**Chronic circadian rhythm disorder induces heart failure with preserved ejection fraction-like phenotype through the Clock-sGC-cGMP-PKG1 signaling pathway**

Yiyang Che, M.D.^1^; Yuuki Shimizu, M.D., Ph.D.^1*^; Takumi Hayashi, M.D.^1^; Junya Suzuki, M.D.^1^; Zhongyue Pu, M.D., Ph.D.^1^; Kazuhito Tsuzuki, M.D., Ph.D.^1^; Shingo Narita, M.D.^1^; Rei Shibata, M.D., Ph.D.^2^; Toyoaki Murohara, M.D., Ph.D.^1^

^1^Department of Cardiology, Nagoya University Graduate School of Medicine, Nagoya 466-8550, Japan

^2^Department of Advanced Cardiovascular Therapeutics, Nagoya University Graduate School of Medicine, Nagoya 466-8550, Japan

*Correspondence to: Yuuki Shimizu, MD, PhD, FESC, FJCS. Department of Cardiology, Nagoya University Graduate School of Medicine, 65 Tsurumai, Showa-ku, Nagoya 466-8550, Japan. Tel: +81-52-744-2147; Fax: +81-52-744-2138; E-mail: [shimi123@med.nagoya-u.ac.jp](mailto:shimi123@med.nagoya-u.ac.jp).


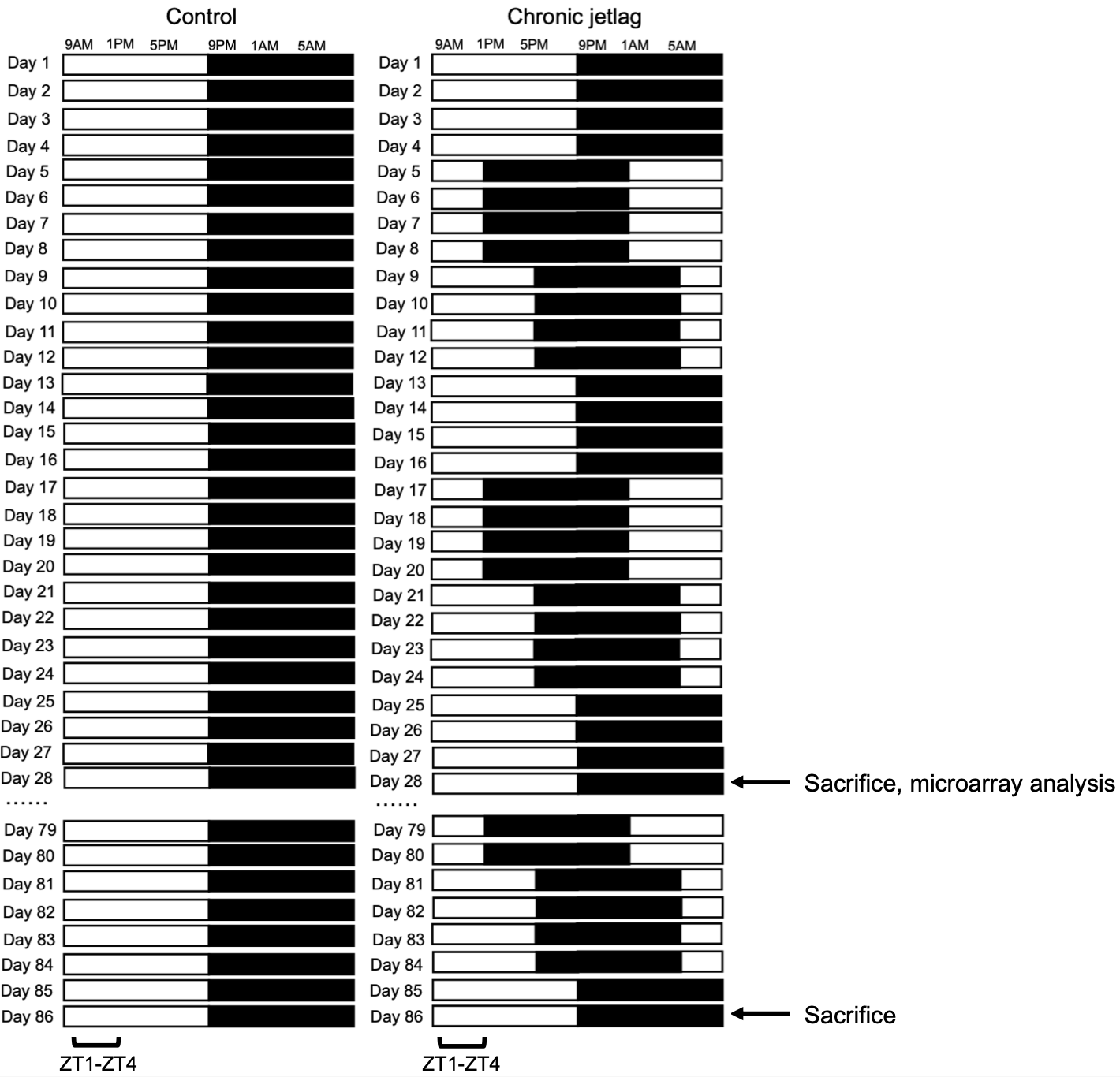


(Echocardiographic and blood pressure measurement at ZT0-ZT6)

**Supplementary Figure S1**

Chronic jetlag lighting schedule. Black bars represent dark phase and hollow bars represent light phase.

**A**

**B**

**C**

**D**


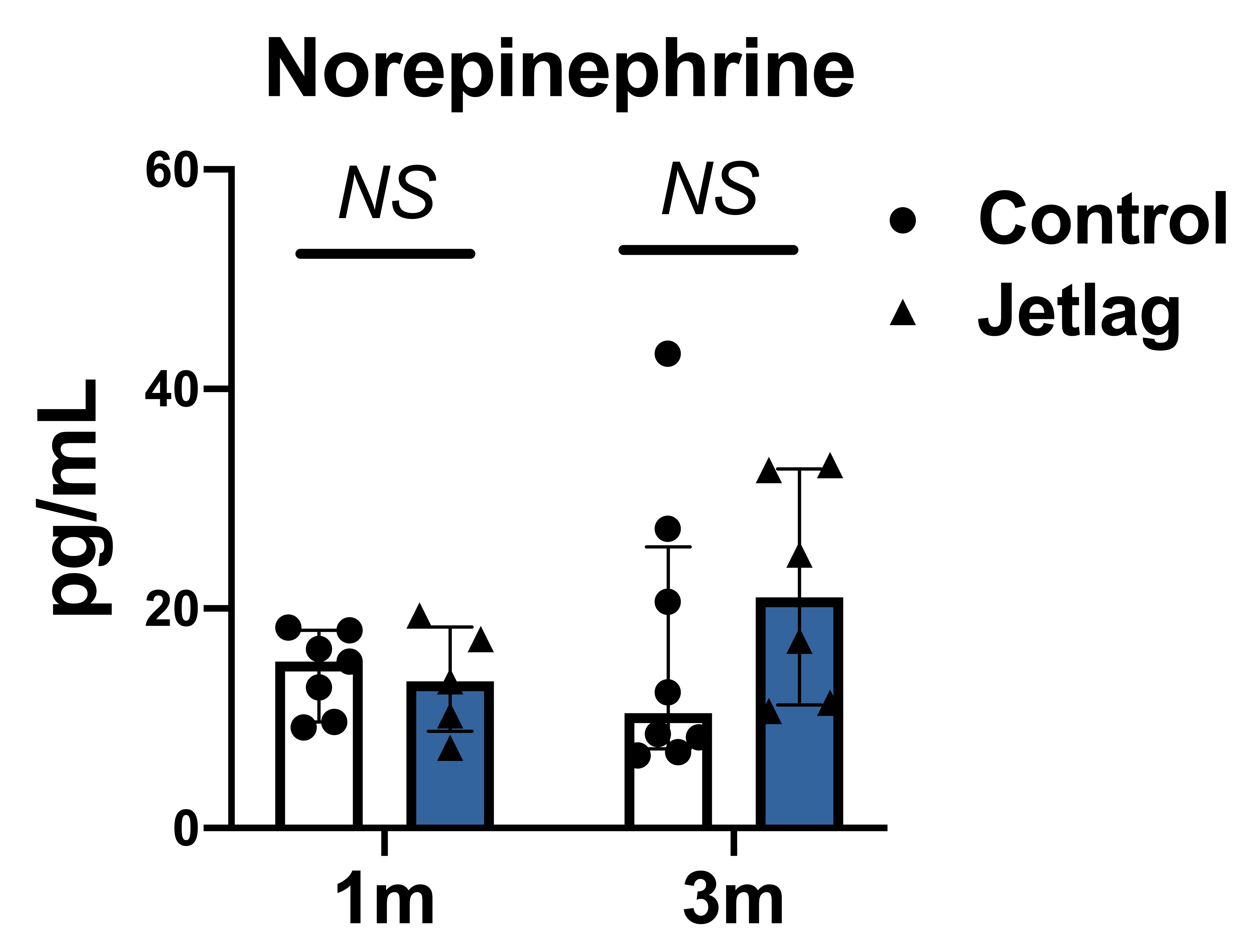

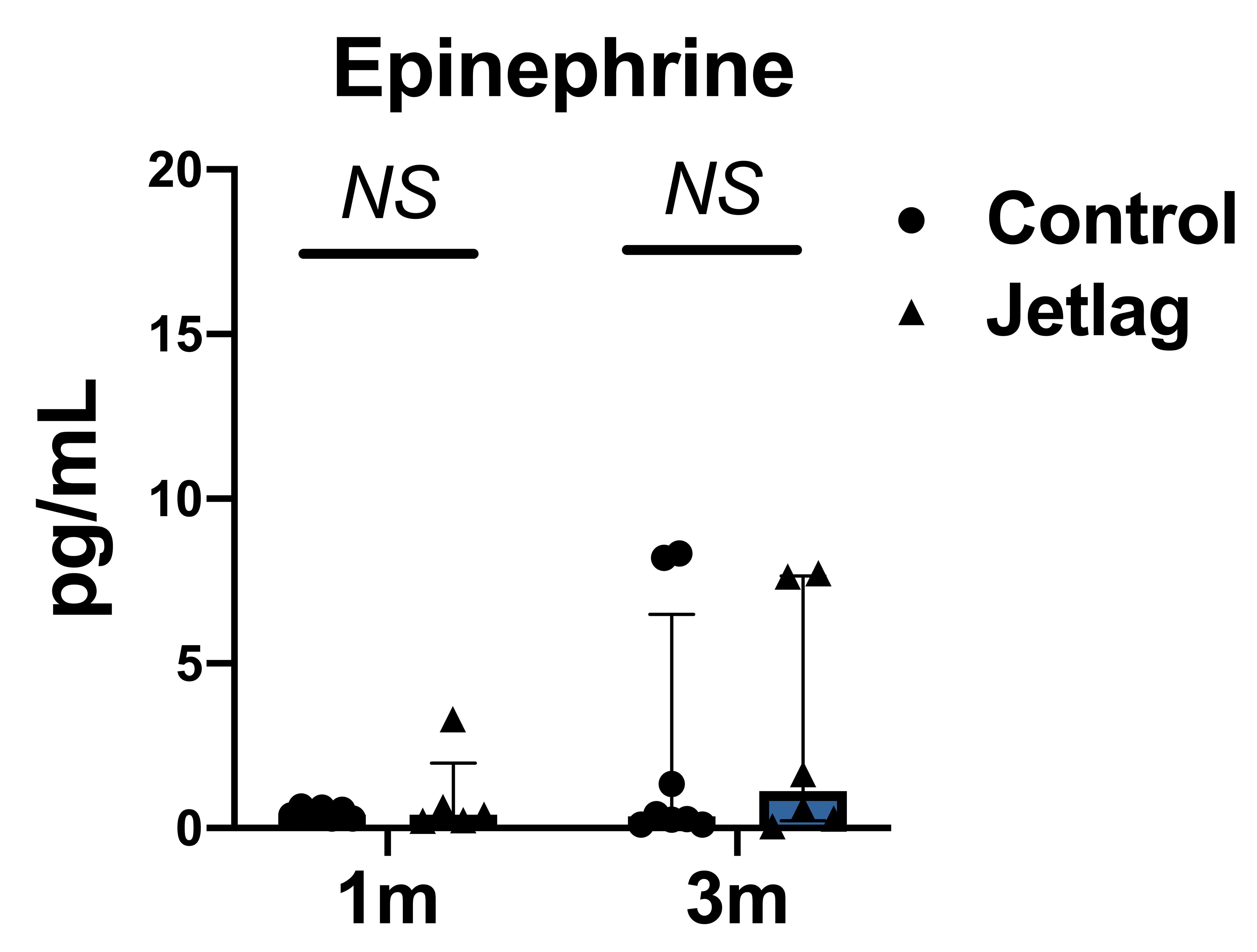

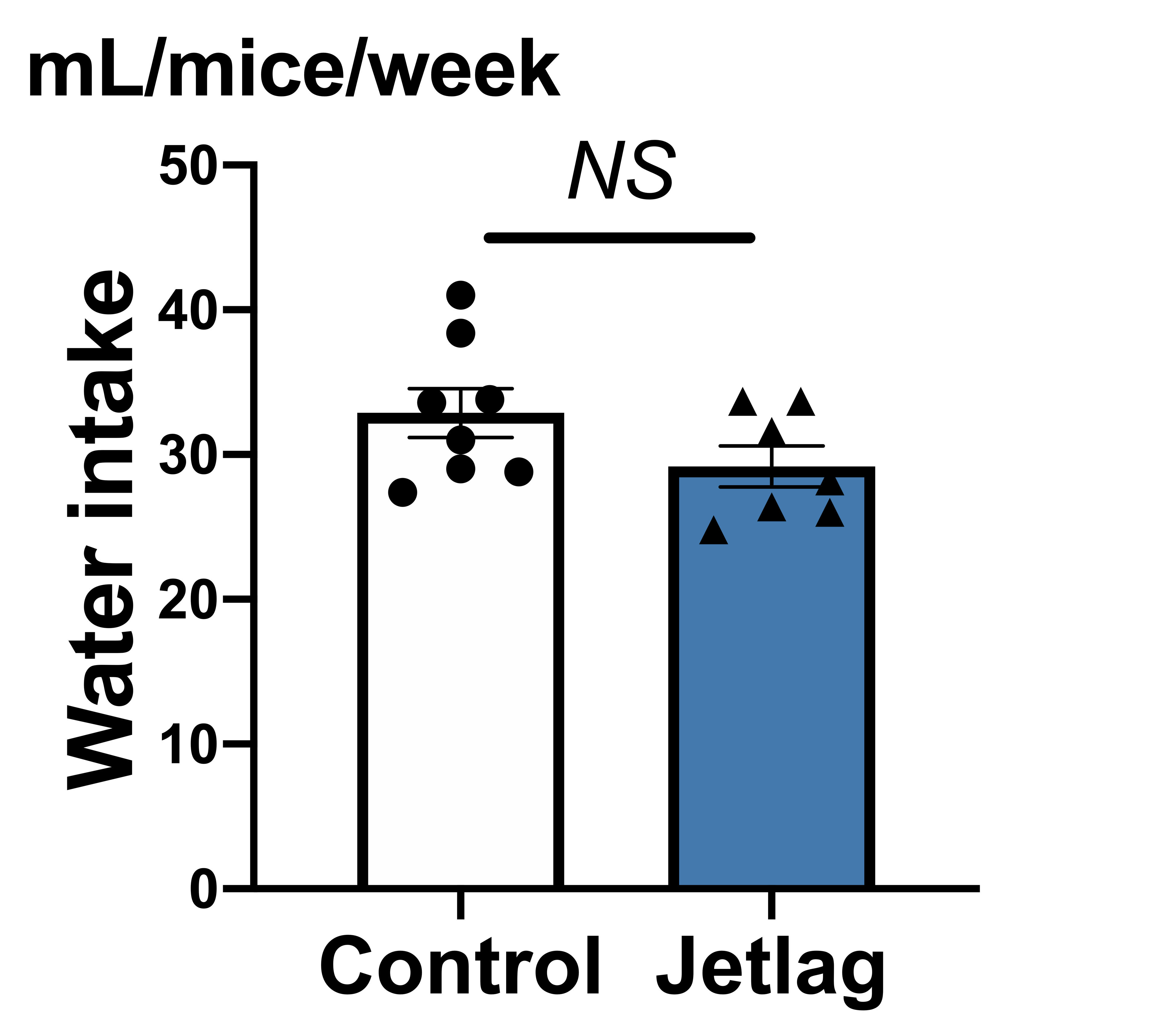

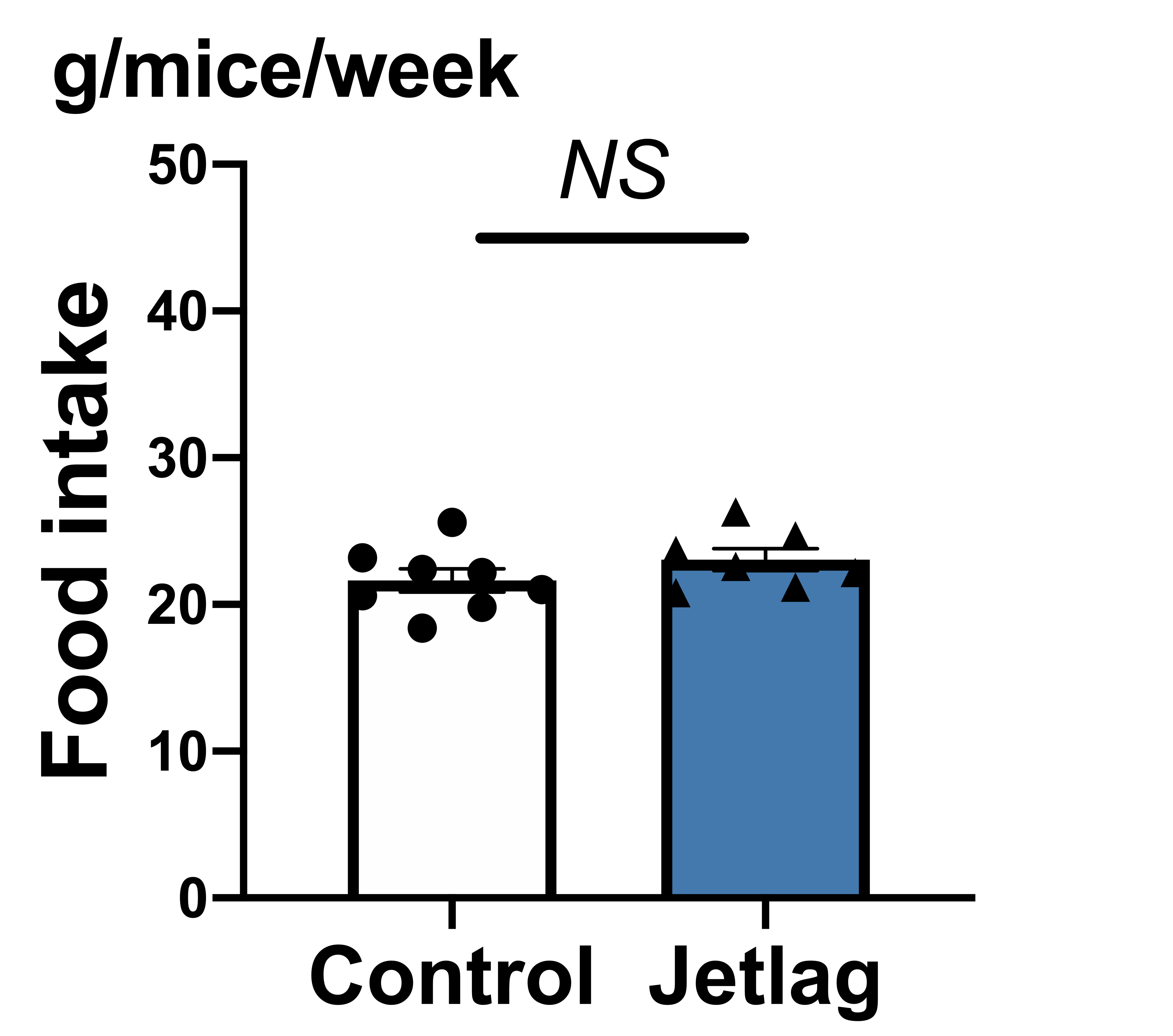


**H**

**G**

**F**

**E**

**3m**


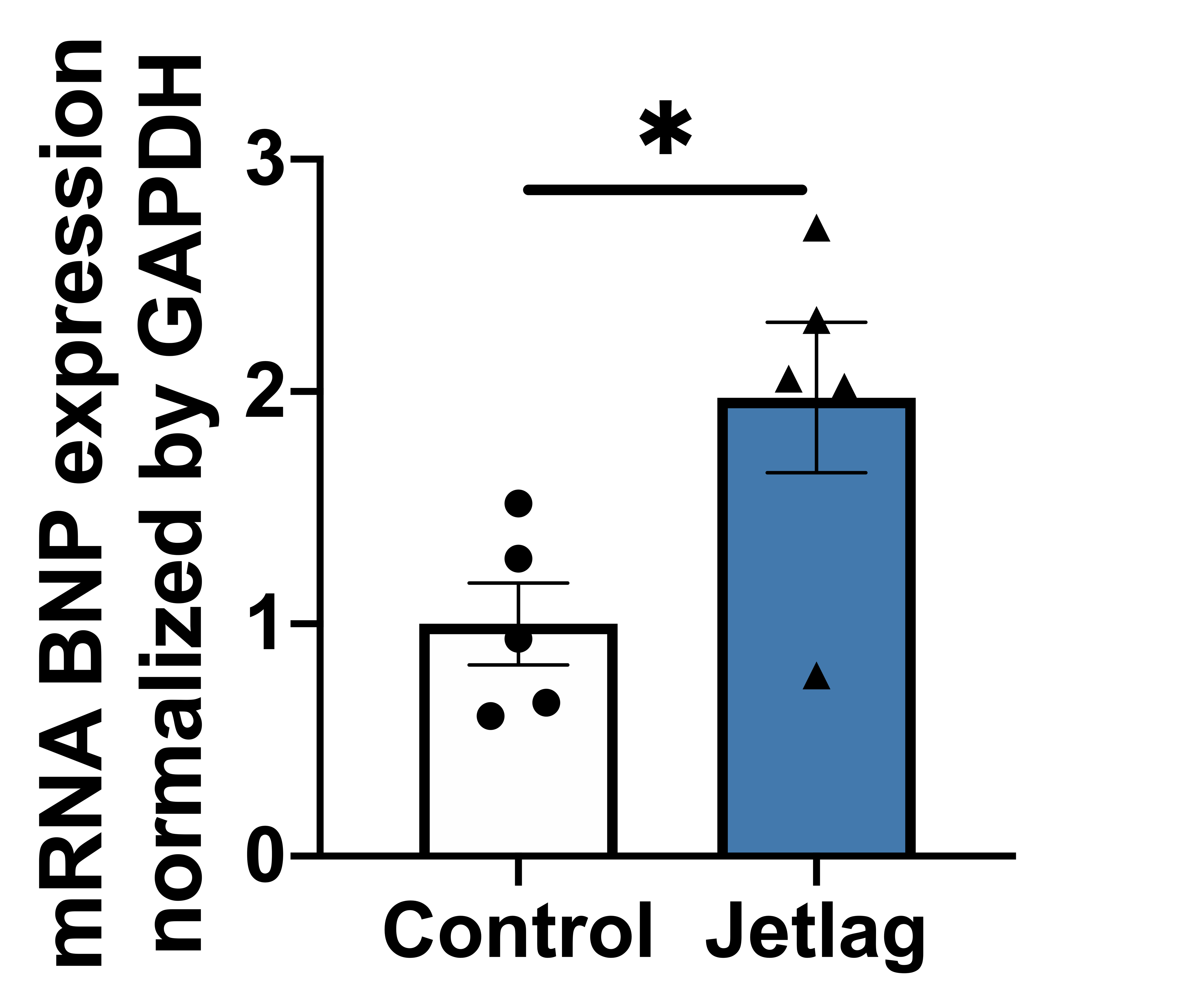

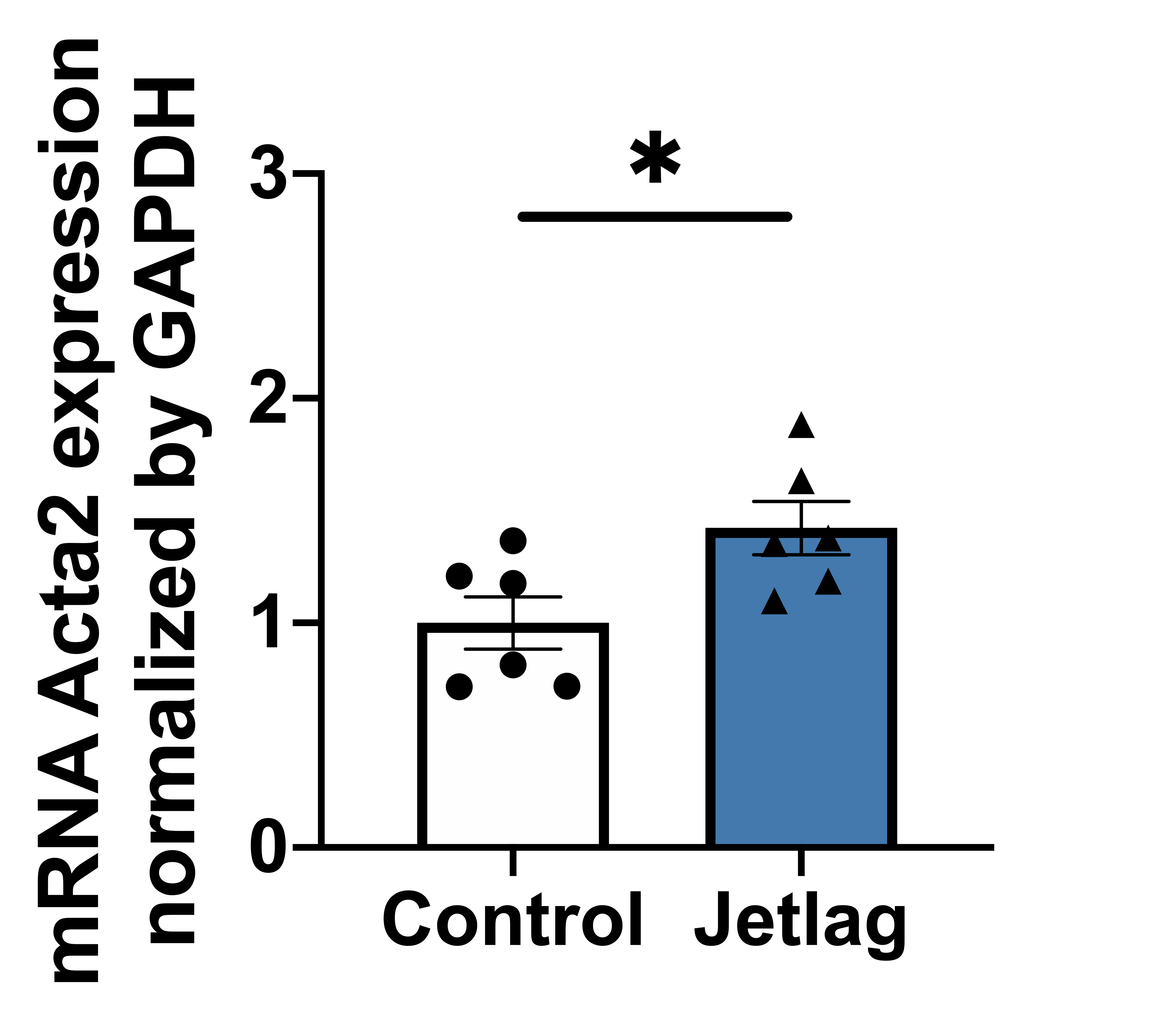

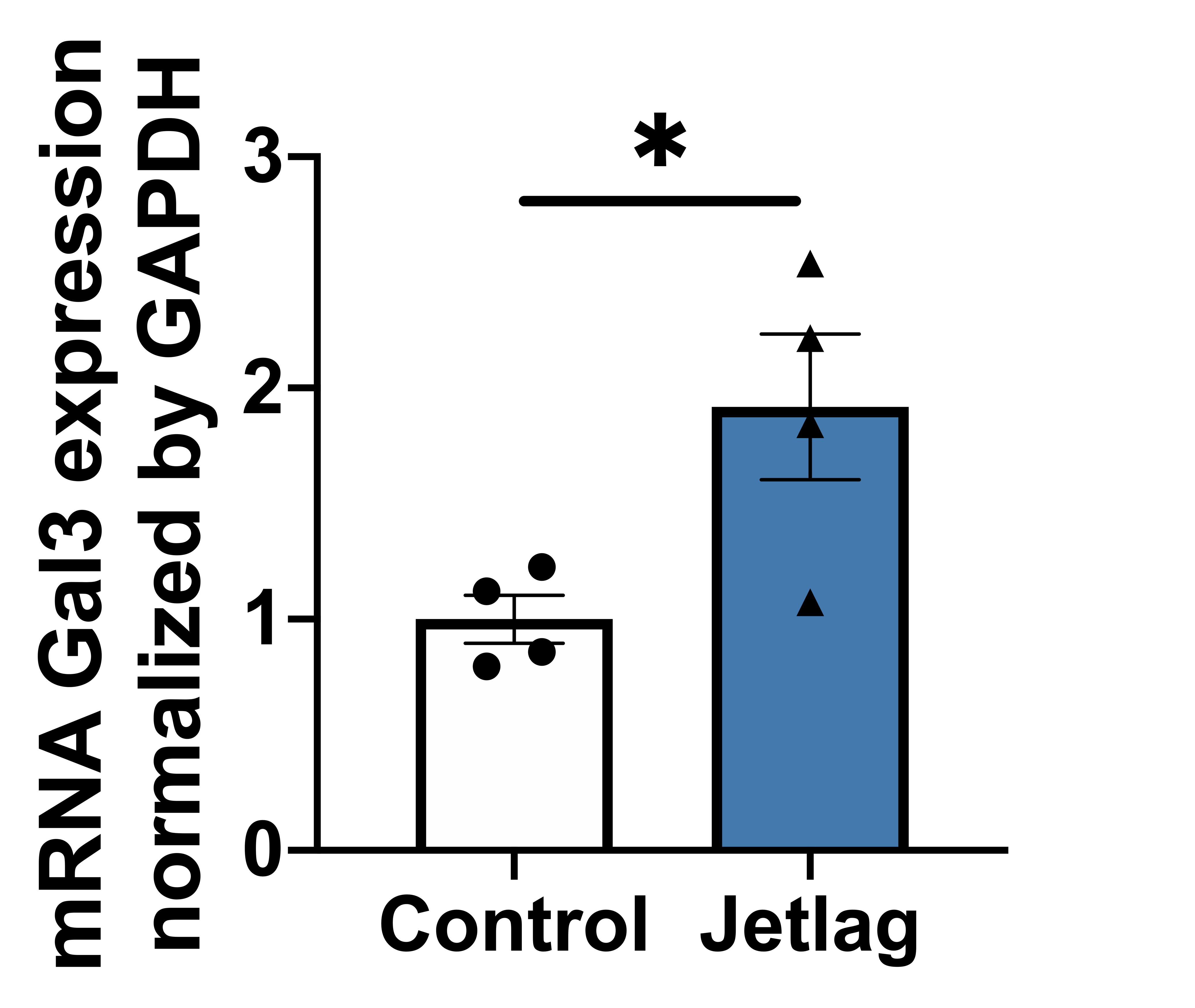

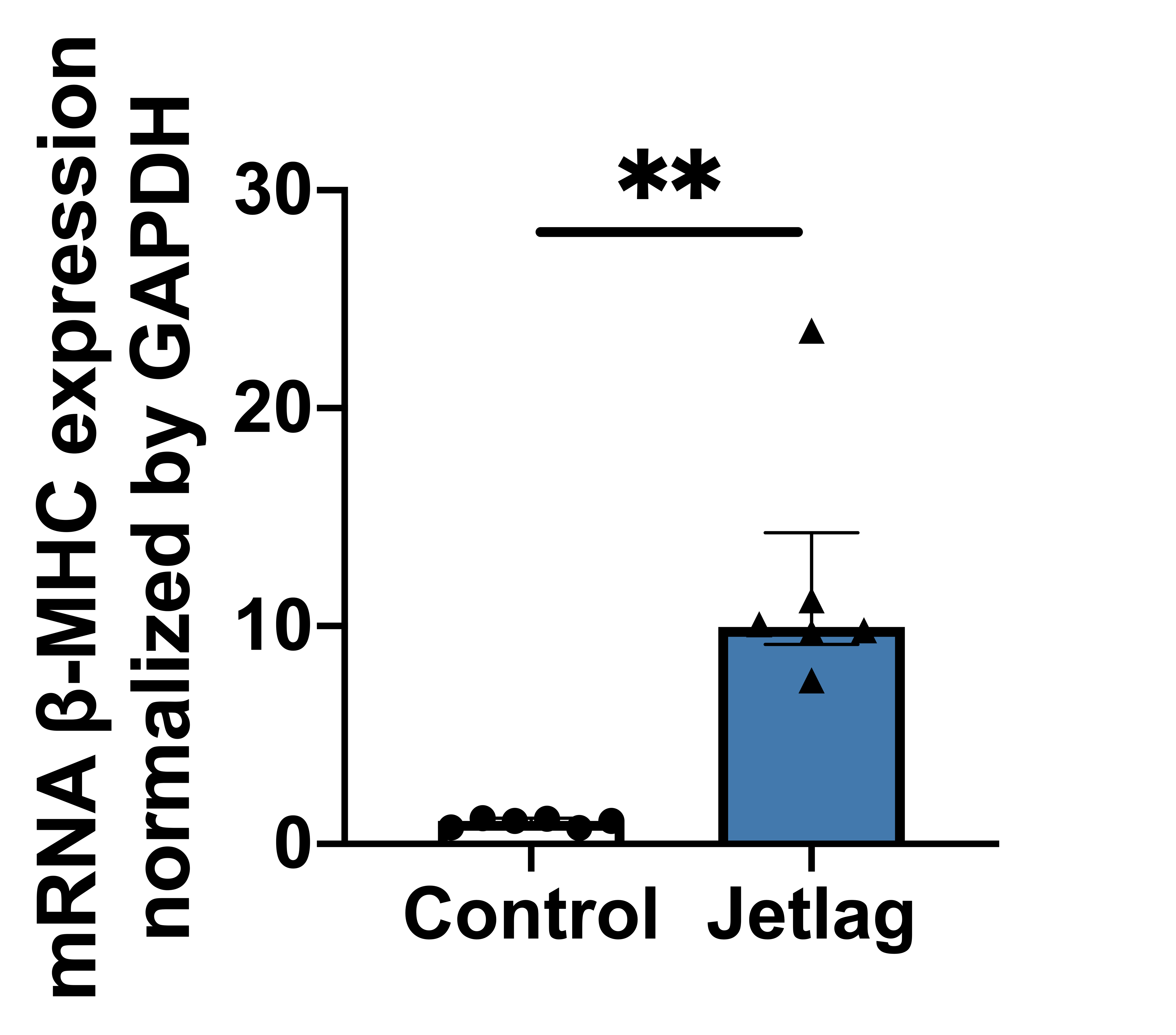


**J**

**I**


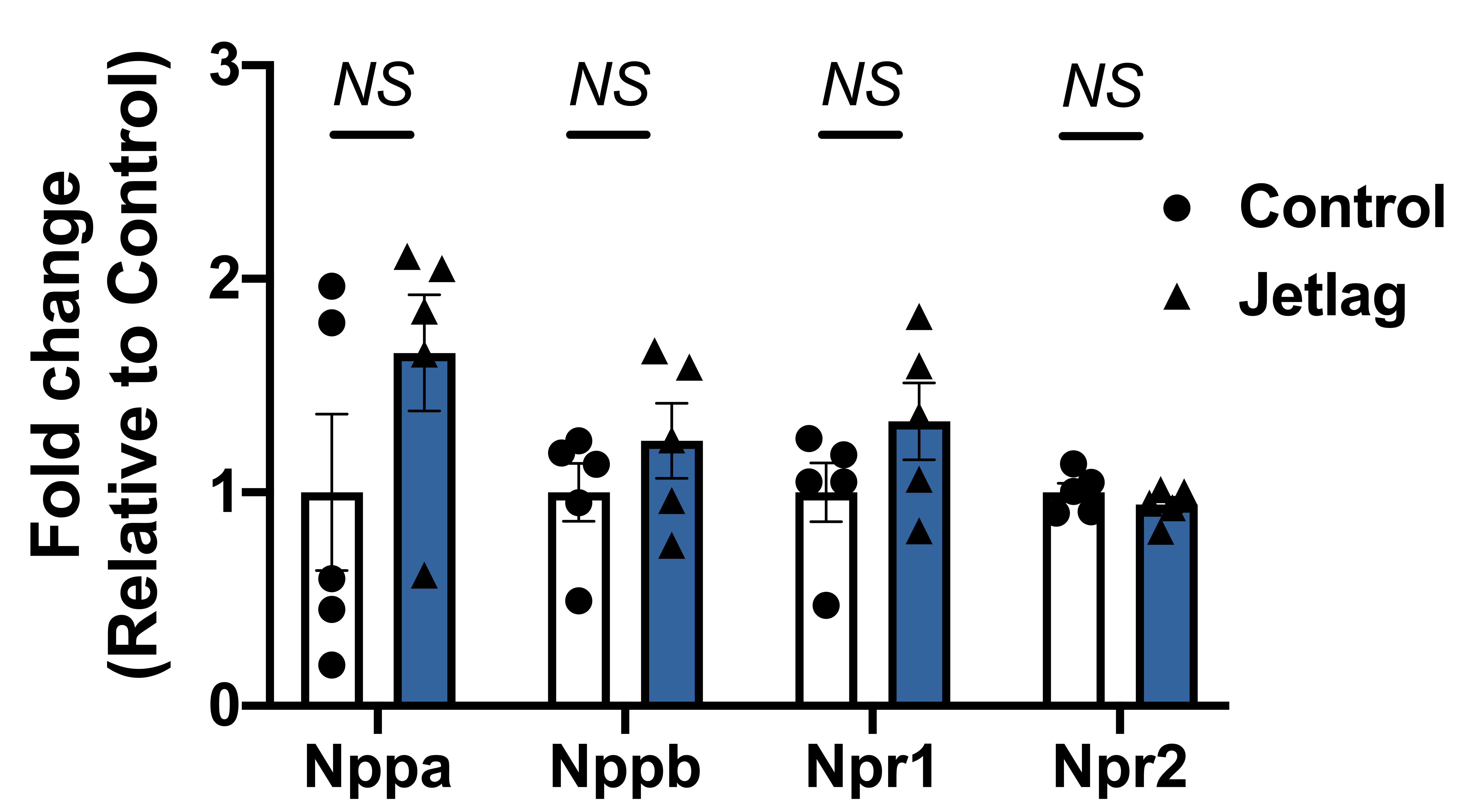

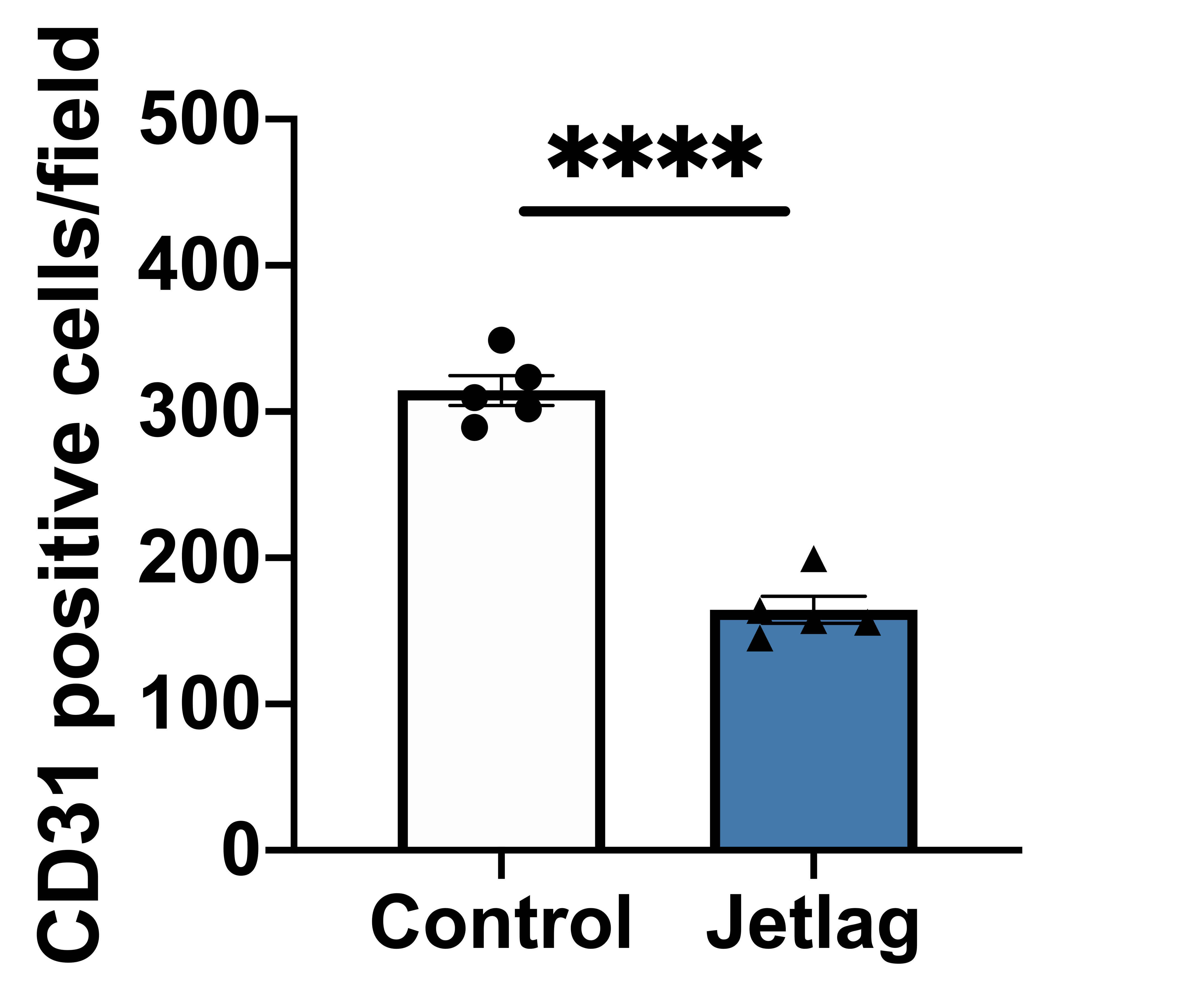

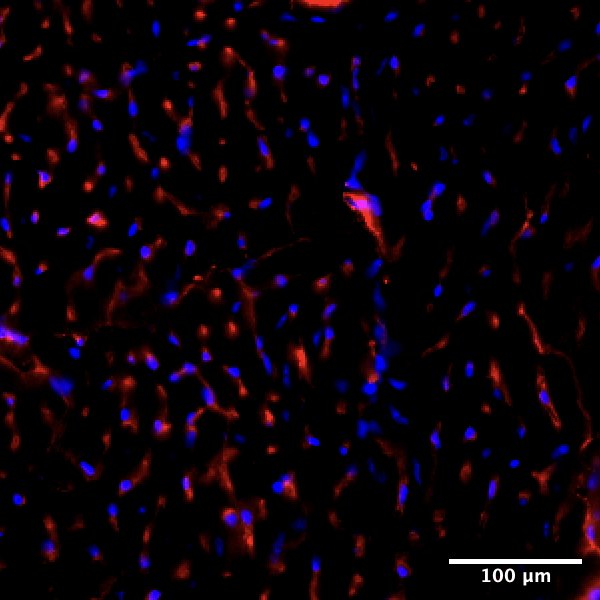

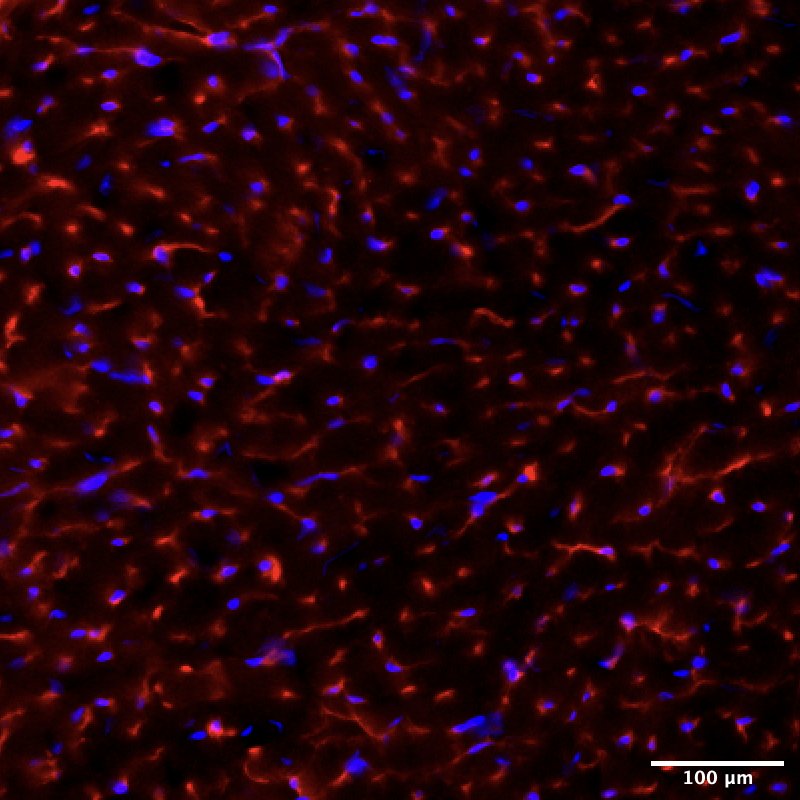


**CD31 DAPI**

**Control**

**Jetlag**

**K**


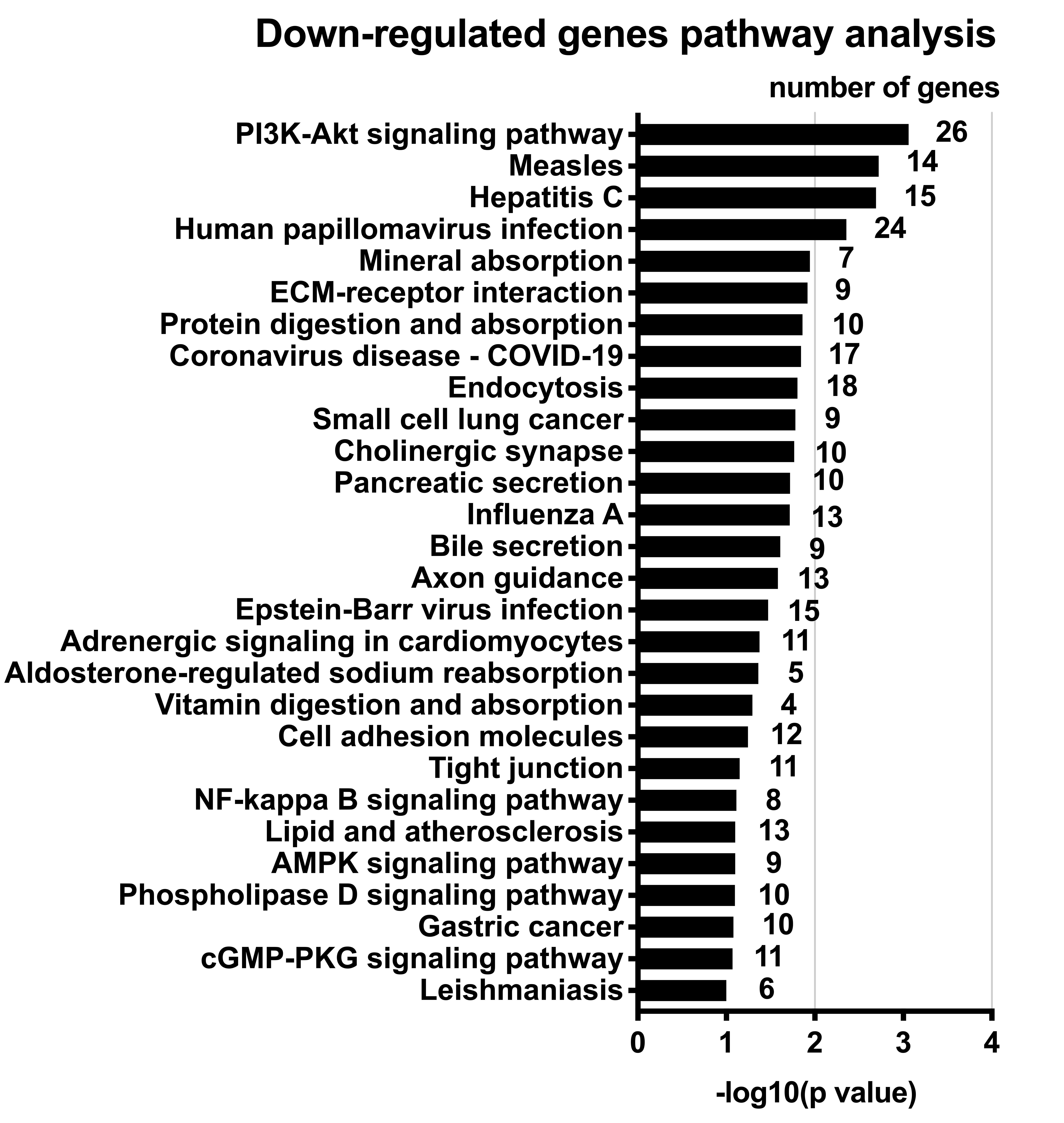


**Supplementary Figure S2**

Cardiac remodeling and signaling in constant jetlag condition. (A) The consumptions of food and (B) water were check in control or 3-month jetlag model for 4 weeks. Five mice were kept in a breeding cage. (control, n=8; jetlag, n=7) (C) Mouse blood serum Epinephrine levels at 1 (control, n=7; jetlag, n=5) and 3 months (control, n=8; jetlag, n=6) of the study. (D) Mouse blood serum Norepinephrine levels at 1 month (control, n=7; jetlag, n=5) and 3 months (control, n=8; jetlag, n=6) of the study. (E) Beta-MHC mRNA expression by RT-qPCR in 1-month control and jet-lagged hearts (n=6 per group). (F) mRNA expression of cardiac fibrosis markers Gal3 (n = 4 per group) and (G) Acta2 (n = 6 per group) by RT-qPCR in 1-month control and jetlag hearts. (H) BNP mRNA expression determined by RT-qPCR in 3-month control and jet-lag hearts (n=5 per group). (I) Representative immunostaining images stained by CD31 (Red) and DAPI (Blue) at 3 months. The white scale bar represents 100µm (20x magnification). And quantification of CD31 positive cells for each group. (n=5 per group) (J) Nppa, Nppb, Npr1, and Npr2 expression levels between the control and jetlag groups are shown using raw data from RNA-seq (n=5 per group). (J) The significantly enriched pathways (P<0.05) in the KEGG pathway analysis of the down-regulated differential expression genes. Data are shown as mean ± SEM and were analyzed by unpaired Student’s t-test (D [1m], F, G, H, I, and J) or median with IQR and analyzed by Mann–Whitney test (C, D [3m], and E). *p<0.05 and **p<0.01 for indicated comparisons.


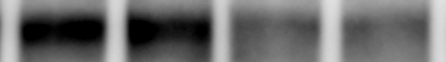

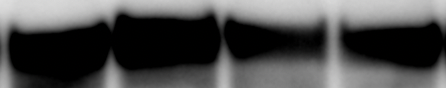


**B**

**A**

**H9c2-siClock**

**H9c2-scr**

**GAPDH**

37kDa

**Clock**

95kDa


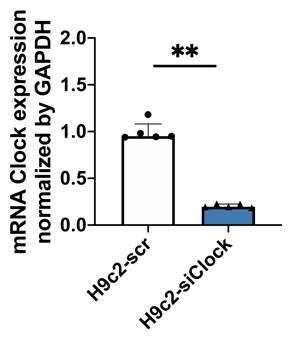

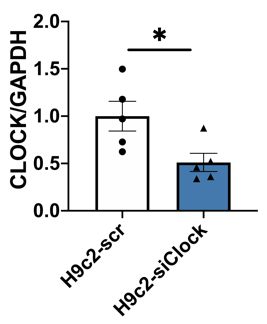


**C**


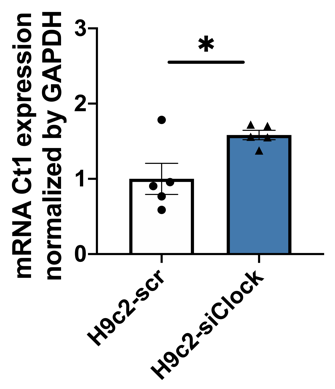

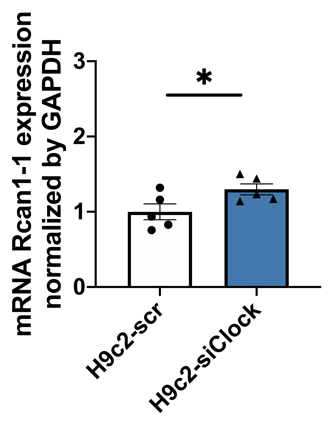

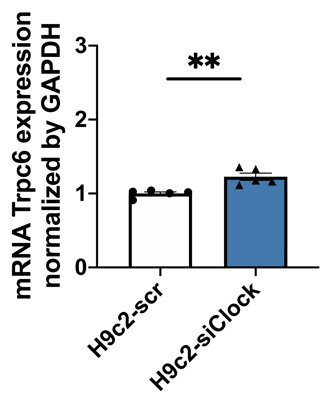


**Supplementary Figure S3**

**Clock gene knock-down efficiency and cardiac hypertrophy markers expression in** H9c2-scr and Clock-KD H9c2 cardiomyocytes. (A) *Clock* mRNA expression determined by qRT-PCR in H9c2-scr and Clock-KD H9c2 cardiomyocytes (n=5 per group). (B) Representative western blots and quantification of CLOCK and GAPDH in H9c2-scr and Clock-KD H9c2 cardiomyocytes (n=5 per group). (C) mRNA expression of cardiac hypertrophy markers *Ct1*, *Rcan1-1*, and *Trpc6* by qRT-PCR in H9c2-scr and Clock-KD H9c2 cardiomyocytes (n=5 per group). Data are shown as mean ± SEM and were analyzed by unpaired Student’s t-test (B [protein CLOCK], and C) or median with IQR and analyzed by Mann–Whitney test (A [mRNA *Clock*]). *p<0.05 and **p<0.01 for indicated comparisons.

**Original blots**

Figure 3C

**Loading**

**Clock**

95kDa

**ZT12**

**ZT6**

**ZT24**

**ZT18**

**J**

**J**

**C**

**J**

**C**

**C**

**J**

**C**


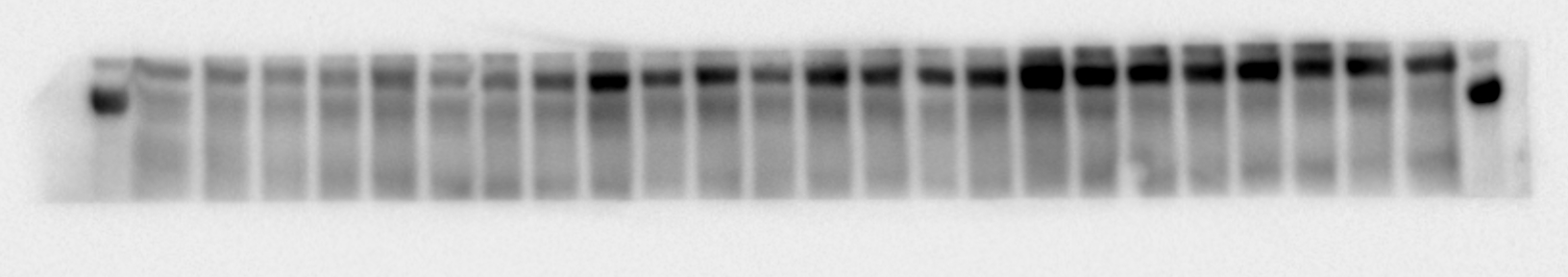


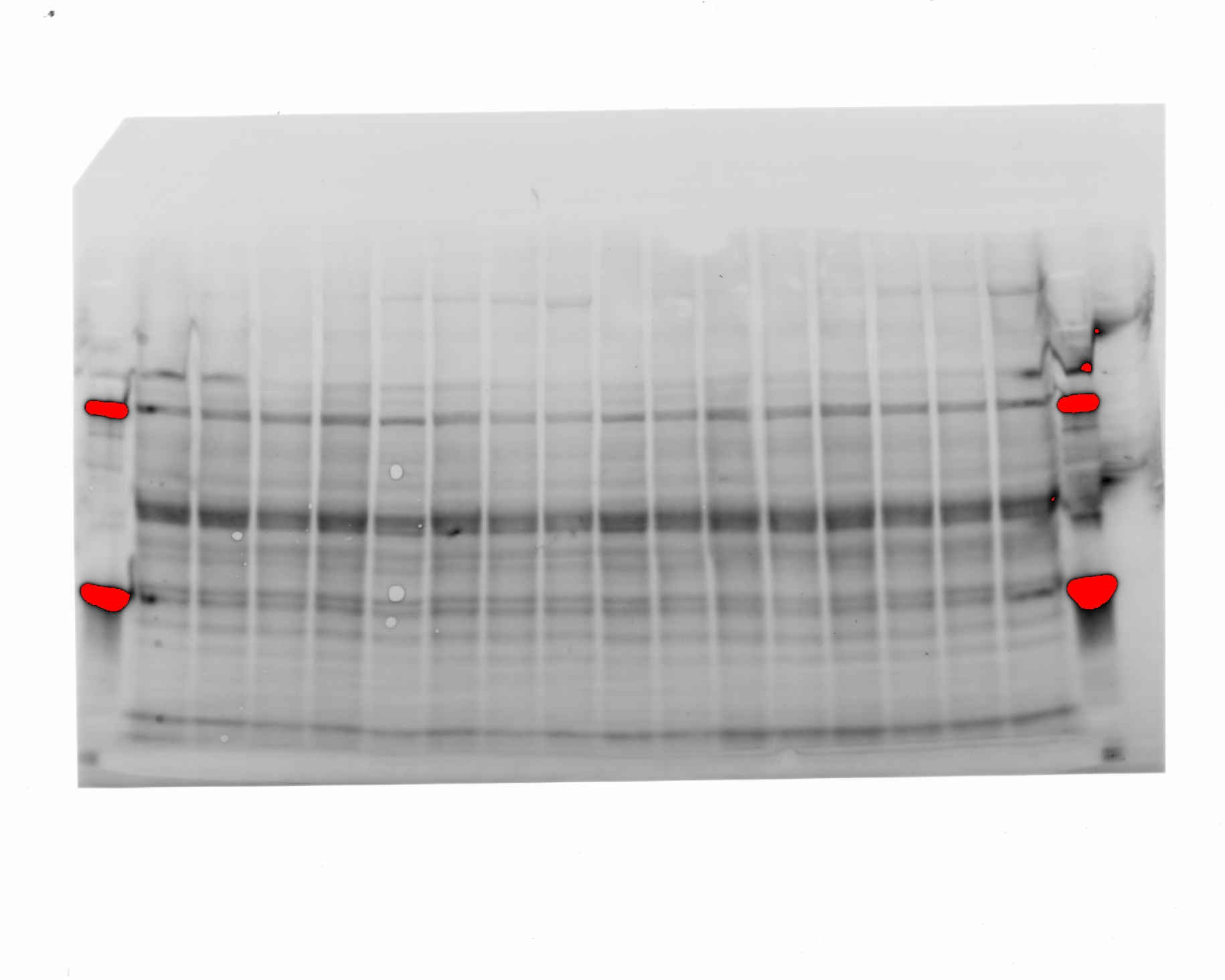


Figure 3H

**Jetlag**

**Control**


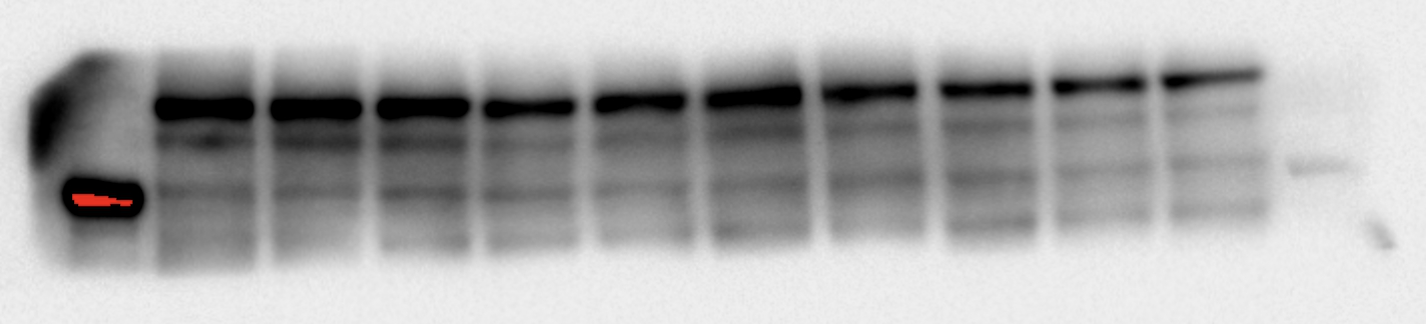


**eNOS**

133kDa


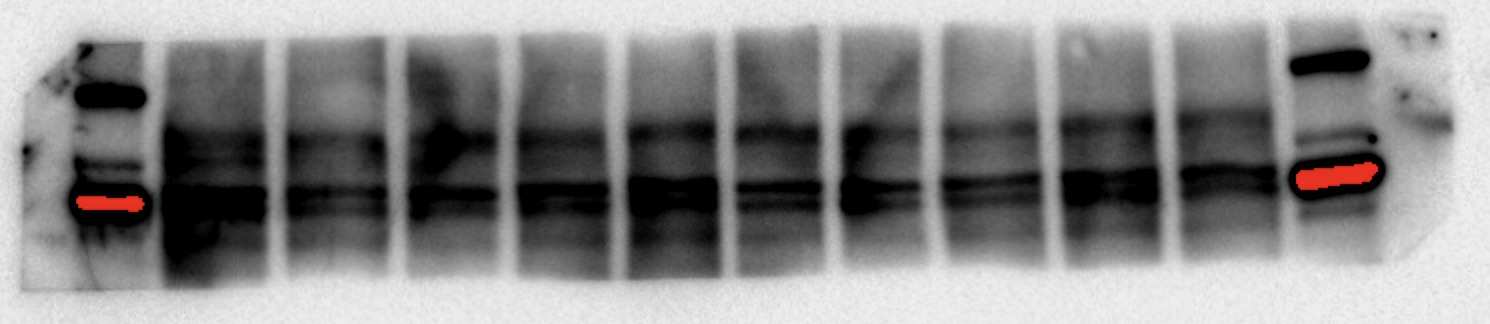


**iNOS**

131kDa


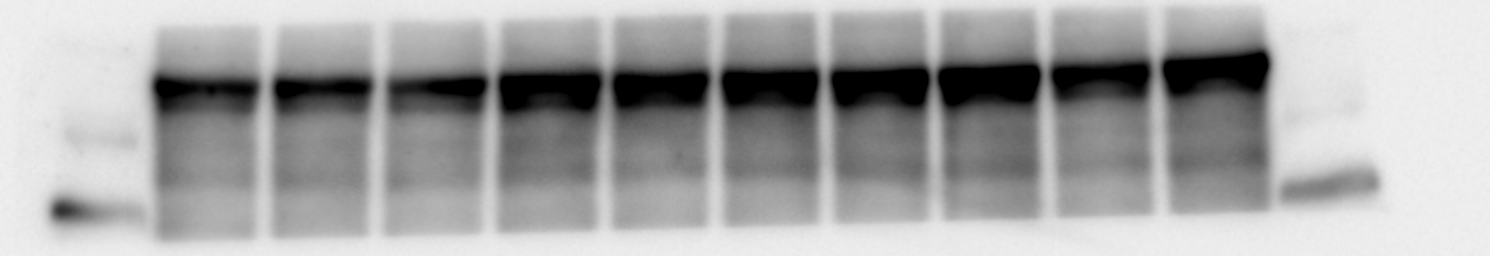

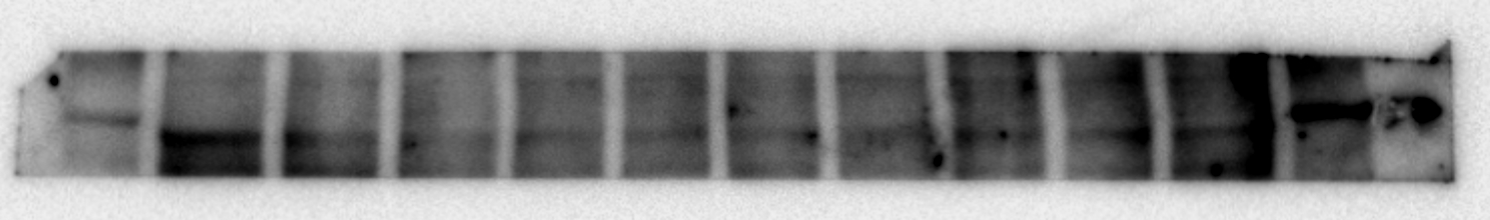


**β-actin**

45kDa

**nNOS**

131kDa

Figure 3I


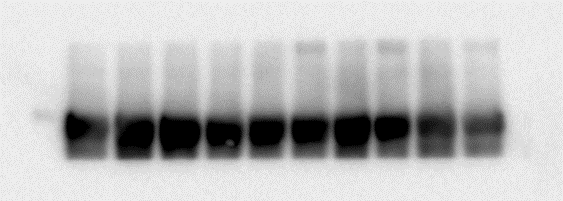


**Jetlag**

**Control**

**PKG1a**

78kDa


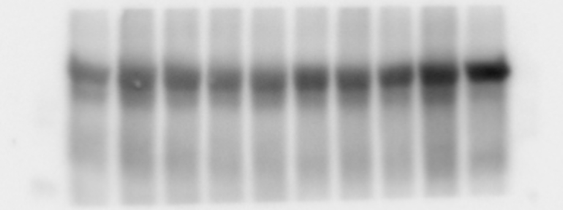


**β-actin**

45kDa

Figure 3J


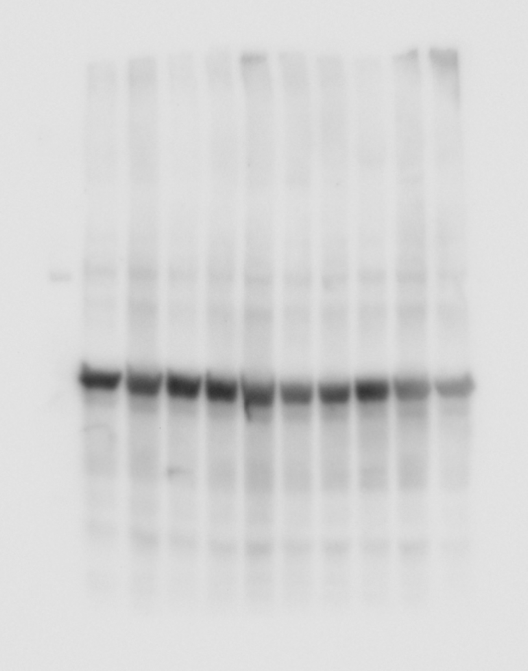

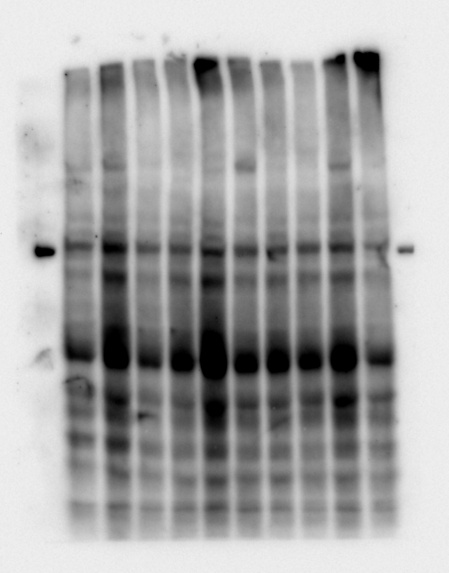


**Control**

**Jetlag**

**Jetlag**

**Control**

250kDa

150kDa

100kDa

75kDa

50kDa

37kDa

25kDa

**TITIN**

**β-actin**

45kDa

Figure 4B


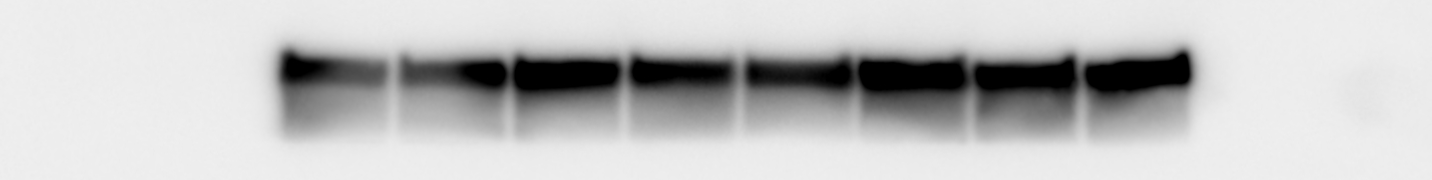

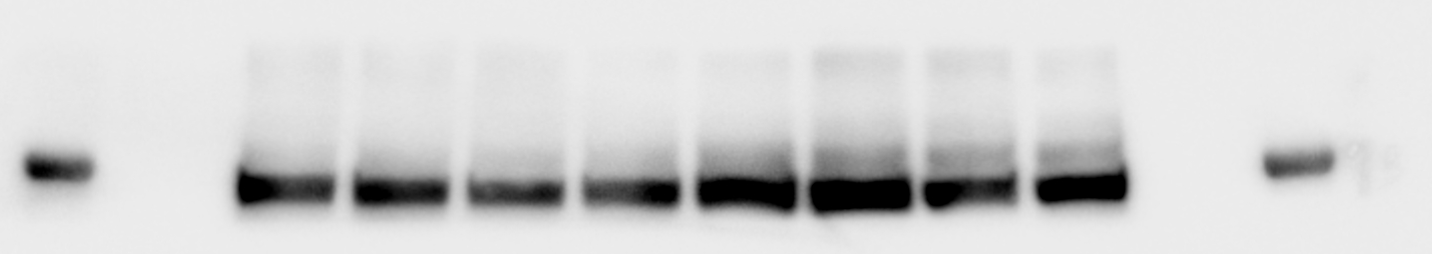

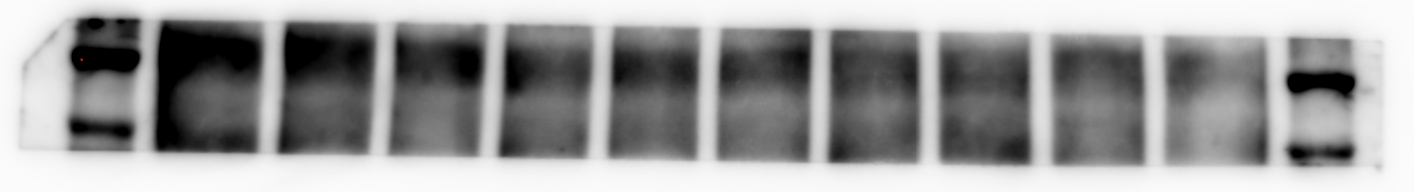

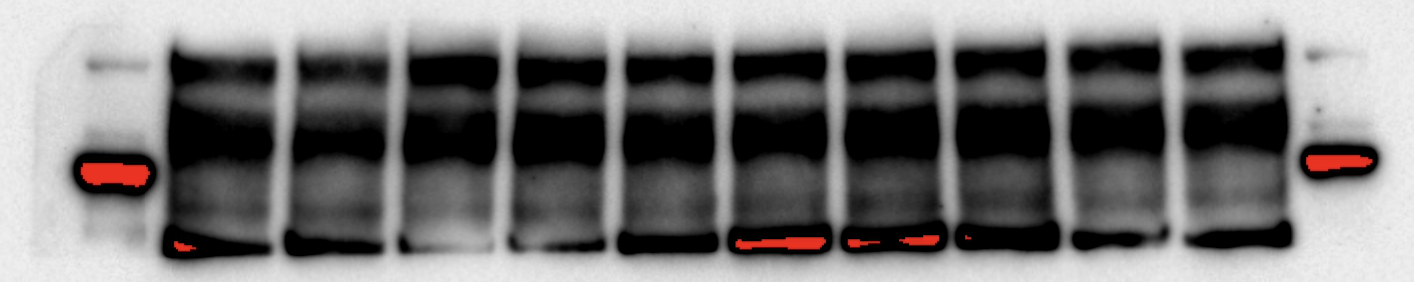


**H9c2-siClock**

**PKG1**

78kDa

**GAPDH**

37kDa

**H9c2-scr**

**nNOS**

130kDa

**GAPDH**

37kDa

**iNOS**

131kDa

**eNOS**

133kDa

**H9c2-scr**

Figure 4E


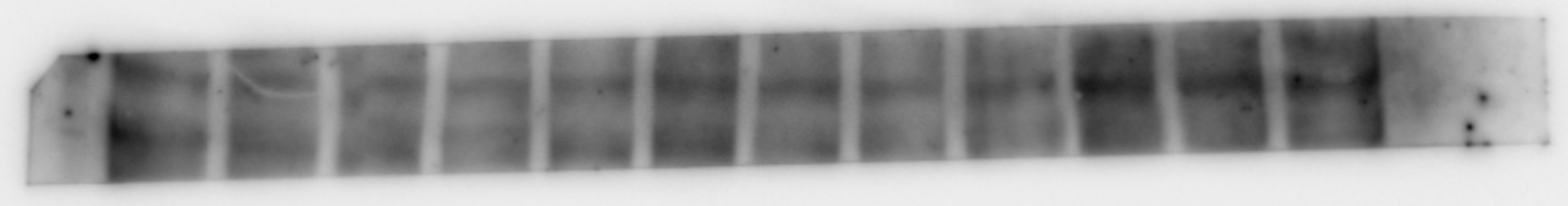


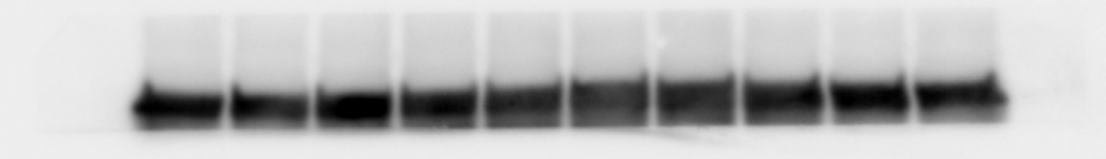


**H9c2-siClock**

Figure 4G

**H9c2-siClock**

**H9c2-scr**


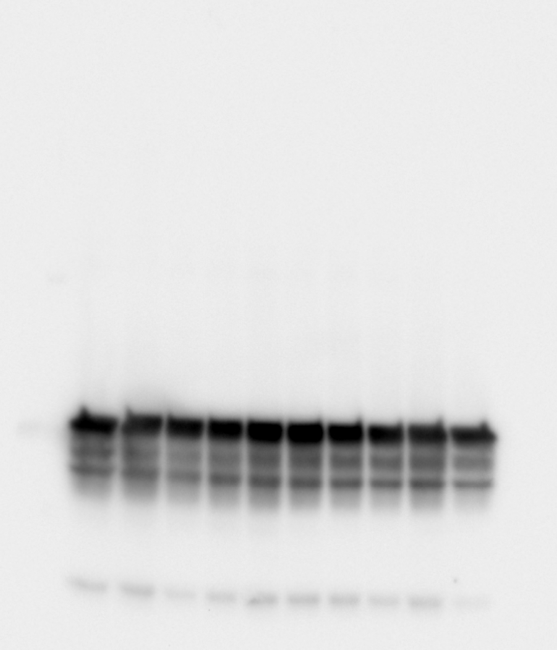

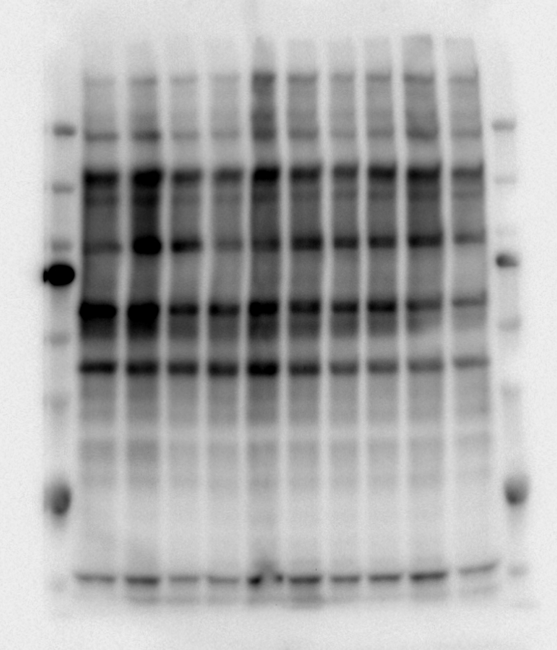


250kDa

150kDa

75kDa

100kDa

**H9c2-scr**

**H9c2-siClock**

50kDa

37kDa

25kDa

**GAPDH**

37kDa

**TITIN**


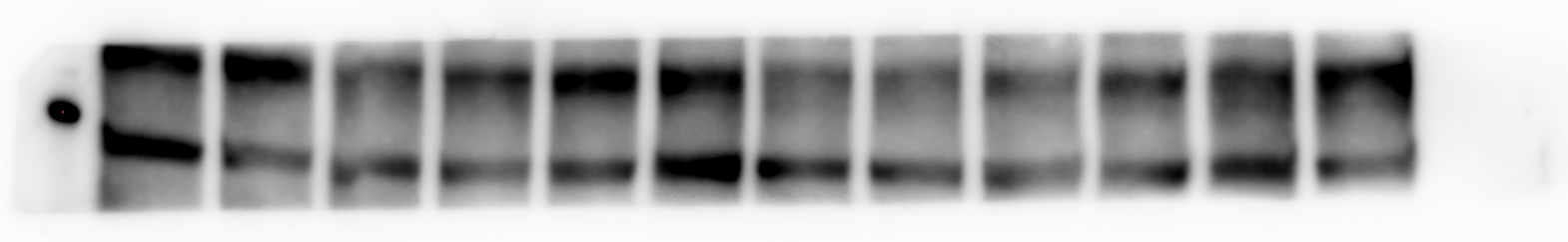

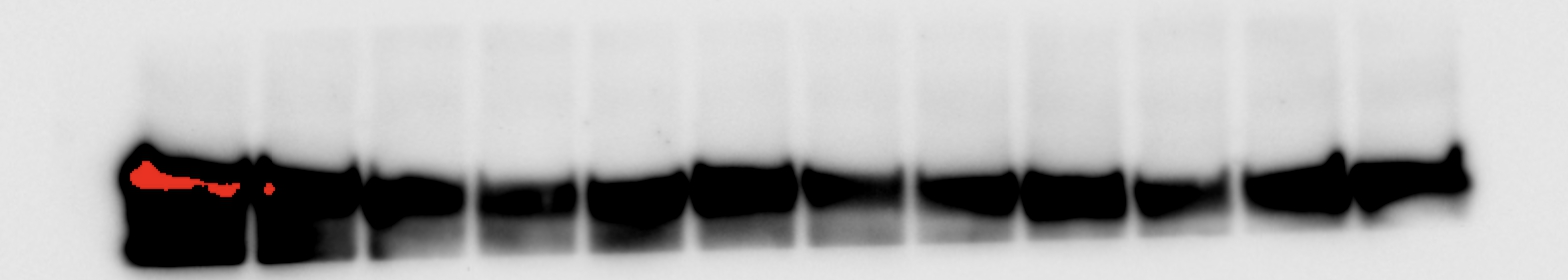


Figure S3B

**Clock**

95kDa

**GAPDH**

37kDa

**H9c2-siClock**

**H9c2-scr**

Table S1. Echocardiography at 1, and 3 months of the study.

|  | Control | Jetlag |
| --- | --- | --- |
| n | 7 | 8 |
| Baseline |  |  |
| LVIDd (mm) | 3.19±0.07 | 3.27±0.08 |
| LVIDs (mm) | 1.78±0.06 | 1.83±0.06 |
| EF (%) | 76.58±1.31 | 76.45±1.26 |
| FS (%) | 44.14±1.24 | 44.11±1.17 |
| IVSd (mm) | 0.89±0.04 | 0.85±0.02 |
| PWTd (mm) | 0.77±0.01 | 0.78±0.02 |
| Corrected LV Mass (mg) | 69.09±3.84 | 69.68±3.34 |
| HR (bpm) | 573.24±13.52 | 549.12±23.23 |
|  |  |  |
| 1 month |  |  |
| LVIDd (mm) | 3.46±0.10 | 3.23±0.12 |
| LVIDs (mm) | 1.89±0.05 | 1.78±0.05 |
| EF (%) | 77.49±1.38 | 77.19±1.31 |
| FS (%) | 45.27±1.31 | 44.61±1.23 |
| IVSd (mm) | 0.70±0.05 | 0.86±0.05* |
| PWTd (mm) | 0.75±0.04 | 1.01±0.05** |
| Corrected LV Mass (mg) | 65.25±3.33 | 83.87±5.28* |
| HR (bpm) | 550.07±21.17 | 565.39±16.99 |
|  |  |  |
| 3 months |  |  |
| LVIDd (mm) | 3.44±0.04 | 3.17±0.06** |
| LVIDs (mm) | 1.88±0.05 | 1.74±0.06 |
| EF (%) | 77.78±1.20 | 77.66±1.45 |
| FS (%) | 45.51±1.18 | 45.20±1.39 |
| IVSd (mm) | 0.80±0.04 | 0.91±0.02* |
| PWTd (mm) | 0.85±0.02 | 1.14±0.05*** |
| Corrected LV Mass (mg) | 77.25±3.53 | 93.45±3.73** |
| HR (bpm) | 569.41±8.62 | 581.29±22.84 |

Echocardiography was measured between ZT0-ZT6 (ZT: zeitgeber time). LVIDd indicates left ventricle internal dimensions at diastole; LVIDs, left ventricle internal dimensions at systole; EF, ejection fraction; FS, fractional shortening; IVSd, septal wall thickness at diastole; PWTd, posterior wall thickness at diastole; HR, heart rate. *p<0.05, **p<0.01, ***p<0.001 significant vs. control. Normally distributed data were analyzed by unpaired two-tailed Student t test, non-normally distributed data were analyzed by Mann-Whitney test. Values are mean ± SEM.

Table S2. Blood Pressure at 1, 2 and 3 months of the study.

|  | Control | Jetlag | Jetlag + RIO |
| --- | --- | --- | --- |
| n | 8 | 8 | 8 |
| Baseline |  |  |  |
| SBP | 107.54±2.36 | 104.04±3.05 | 109.92±1.83 |
| MBP | 83.96±1.46 | 83.67±2.27 | 88.13±1.31 |
| DBP | 72.29±1.88 | 72.29±1.94 | 77.25±1.28 |
| HR | 643.79±11.48 | 601.67±25.41 | 645.63±10.42 |
|  |  |  |  |
| 1 month |  |  |  |
| SBP | 102.29±2.23 | 116.58±12.47*** | 104.56±12.35^ƗƗ^ |
| MBP | 84.71±1.85 | 90.67±9.67* | 84.63±10.03^Ɨ^ |
| DBP | 76.04±1.79 | 81.29±8.66 | 75.73±9.10 |
| HR | 628.83±12.55 | 608.88±64.50 | 618.13±73.24 |
|  |  |  |  |
| 2 months |  |  |  |
| SBP | 102.83±1.89 | 118.88±2.23*** | 105.17±3.06 ^ƗƗ^ |
| MBP | 85.75±2.67 | 95.17±2.56* | 86.92±2.47 |
| DBP | 77.25±3.14 | 83.42±3.09 | 77.88±2.39 |
| HR | 617.00±19.51 | 608.83±11.85 | 627.83±22.68 |
|  |  |  |  |
| 3 months |  |  |  |
| SBP | 100.46±1.42 | 123.50±3.64**** | 111.00±2.78^Ɨ^ |
| MBP | 78.75±2.27 | 93.83±2.84*** | 87.31±1.75 |
| DBP | 67.92±3.27 | 79.08±2.71* | 75.60±1.63 |
| HR | 638.04±15.07 | 625.50±12.70 | 635.23±14.54 |

Blood pressures were measured between ZT0-ZT6 (ZT: zeitgeber time).HR indicates heart rate; SBP, systolic blood pressure; MBP, mean blood pressure; DBP, diastolic blood pressure. *p<0.05, **p<0.01, ***p<0.001, ****p<0.0001 significant vs. control, ^Ɨ^p<0.05, ^ƗƗ^p<0.01 significant vs. Jetlag. Normally distributed data were analyzed by one-way ANOVA, with Tukey’s multiple comparisons test; and non-normally distributed data were analyzed by Kruskall-Wallis test, with Dunn multiple comparisons test. Values are mean ± SEM.

Table S3. Echocardiography at 1, 2 and 3 months of the study.

|  | Control | Jetlag | Jetlag + RIO |
| --- | --- | --- | --- |
| n | 8 | 8 | 8 |
| Baseline |  |  |  |
| LVIDd (mm) | 3.04±0.09 | 3.10±0.08 | 3.12±0.08 |
| LVIDs (mm) | 1.61±0.05 | 1.63±0.04 | 1.64±0.05 |
| EF (%) | 80.00±0.06 | 80.19±0.39 | 80.04±0.78 |
| FS (%) | 47.23±0.70 | 47.46±0.43 | 47.38±0.78 |
| IVSd (mm) | 0.91±0.02 | 0.88±0.04 | 0.90±0.03 |
| PWTd (mm) | 0.89±0.02 | 0.88±0.01 | 0.87±0.02 |
| Corrected LV Mass (mg) | 72.51±3.68 | 71.29±2.39 | 72.85±2.42 |
| HR (bpm) | 529.59±6.07 | 547.77±13.34 | 544.73±18.16 |
|  |  |  |  |
| 1 month |  |  |  |
| LVIDd (mm) | 3.08±0.10 | 3.03±0.07 | 3.17±0.06 |
| LVIDs (mm) | 1.65±0.07 | 1.59±0.05 | 1.60±0.04 |
| EF (%) | 79.43±1.10 | 80.22±1.07 | 82.21±0.70 |
| FS (%) | 46.78±1.11 | 47.51±1.03 | 49.71±0.74 |
| IVSd (mm) | 0.86±0.03 | 0.99±0.01** | 0.83±0.03^ƗƗ^ |
| PWTd (mm) | 0.86±0.02 | 1.03±0.03*** | 0.83±0.02^ƗƗƗƗ^ |
| Corrected LV Mass (mg) | 69.62±4.88 | 85.29±4.08* | 68.84±3.33^Ɨ^ |
| HR (bpm) | 555.22±18.88 | 537.74±20.32 | 545.15±11.63 |
|  |  |  |  |
| 2 months |  |  |  |
| LVIDd (mm) | 3.27±0.05 | 3.18±0.08 | 3.09±0.13 |
| LVIDs (mm) | 1.76±0.05 | 1.68±0.06 | 1.62±0.09 |
| EF (%) | 78.76±1.15 | 79.81±1.10 | 80.61±1.05 |
| FS (%) | 46.31±1.13 | 47.27±1.12 | 47.97±1.00 |
| IVSd (mm) | 0.84±0.01 | 0.97±0.04** | 0.87±0.03^Ɨ^ |
| PWTd (mm) | 0.83±0.02 | 0.98±0.04**** | 0.89±0.02^ƗƗ^ |
| Corrected LV Mass (mg) | 72.36±2.24 | 86.09±1.40* | 72.08±5.78^Ɨ^ |
| HR (bpm) | 532.62±11.65 | 561.34±21.41 | 568.51±19.48 |
|  |  |  |  |
| 3 months |  |  |  |
| LVIDd (mm) | 3.22±0.10 | 2.99±0.03 | 3.20±0.06 |
| LVIDs (mm) | 1.65±0.08 | 1.55±0.03 | 1.54±0.05 |
| EF (%) | 81.17±1.25 | 80.78±1.01 | 84.06±1.01 |
| FS (%) | 48.74±1.25 | 48.05±1.06 | 51.92±1.17 |
| IVSd (mm) | 0.90±0.02 | 1.09±0.04*** | 0.92±0.02^ƗƗ^ |
| PWTd (mm) | 0.89±0.02 | 1.11±0.03**** | 0.88±0.02^ƗƗƗƗ^ |
| Corrected LV Mass (mg) | 78.27±4.21 | 95.62±3.95* | 77.87±1.79^Ɨ^ |
| HR (bpm) | 542.00±12.18 | 575.01±10.48 | 517.81±29.37 |

Echocardiography was measured between ZT0-ZT6 (ZT: zeitgeber time). LVIDd indicates left ventricle internal dimensions at diastole; LVIDs, left ventricle internal dimensions at systole; EF, ejection fraction; FS, fractional shortening; IVSd, septal wall thickness at diastole; PWTd, posterior wall thickness at diastole; HR, heart rate. *p<0.05, **p<0.01, ***p<0.001, ****p<0.0001 significant vs. control, ^Ɨ^p<0.05, ^ƗƗ^p<0.01, ^ƗƗƗƗ^p<0.0001 significant vs. Jetlag. Normally distributed data were analyzed by one-way ANOVA, with Tukey’s multiple comparisons test; and non-normally distributed data were analyzed by Kruskall-Wallis test, with Dunn multiple comparisons test. Values are mean ± SEM.

Table S4. Circadian rhythm related GO terms in the biological process enrichment analyses by Limma package

| GO term | Gene count | P Value | Fold Enrichment |
| --- | --- | --- | --- |
| GO: 0048511  Rhythmic process | 17 | 0.00696528 | 2.10493827 |
| GO: 0007623  Circadain rhythm | 13 | 0.0074049 | 2.41448802 |

Table S5. Mouse and rat primers were used for qRT-PCR.

| Primer | Forward | Reverse |
| --- | --- | --- |
| *Mouse* |  |  |
| *Acta2* | TCAGCGCCTCCAGTTCCT | AAAAAAAACCACGAGTAACAAATCAA |
| *β-MHC* | TTGGATGAGCGACTCAAAAA | GCTCCTTGAGCTTCTTCTGC |
| *BNP* | CAAGGCCTCACAAAAGAACA | ATCCGATCCGGTCTATCTTG |
| *Gal3* | TTGAAGCTGACCACTTCAAGGTT | AGGTTCTTCATCCGATGGTTGT |
| *GAPDH* | ACCCAGAAGACTGTGGATGG | CACATTGGGGGTAGGAACAC |
| *Nos1* | ACTGACACCCTGCACCTGAAGA | GTGCGGACATCTTCTGACTTCC |
| *Nos2* | CAGCTGGGCTGTACAAACCTT | CATTGGAAGTGAAGCGGTTCG |
| *Nos3* | CCTCGAGTAAAGAACTGGGAAGTG | AACTTCCTTGGAAACACCAGGG |
| *Prkg1a* | ATCCGAGAGGTCGAAGGATCT | ATTCCACGGGGTACATACAGT |
|  |  |  |
| *Rat* |  |  |
| *Clock* | TTCGATCACAGCCCAACTCC | ACCTCCGCTGTGTCATCTTCTC |
| *Ct1* | CAGTGTCCTTCTCTTCATACAGCT | TGTGCTCTTTAGGACCGTTGGGCT |
| *GAPDH* | ATGACTCTACCCACGGCAAG | CTGGAAGATGGTGATGGGTT |
| *Nos1* | GAATACCAGCTGATCCATGGAAC | TCCTCCAGGAGGGTGTCCACCGCATG |
| *Nos2* | CATTCAGATCCCGAAACGTAC | AGCCTCATGGTGAACACGTTCT |
| *Nos3* | TACGGAGCAGCAAATCCAC | GATCAAAGGACTGCAGCCTG |
| *Rcan1-1* | ACTGCGAGATGGAGGAGGTG | CGTCCTGAAGAGGGATTCAAA |
| *Trpc6* | GATATCTTCAAATTCATGGTCATA | ATCCGCATCATCCTCAATTTC |

Table S6. Antibodies used for the experiments.

| Target antigen | Vendor | Catalog number | Working concentration |
| --- | --- | --- | --- |
| β-Actin | Cell Signaling | 3700S | 1:1000 |
| CLOCK | Abcam | ab178525 | 0.2µg/mL |
| eNOS | Abcam | ab5589 | 1:1000 |
| GAPDH | Cell Signaling | 2118S | 1:1000 |
| iNOS | Abcam | ab178945 | 1:1000 |
| nNOS | Abcam | ab3511 | 1:1000 |
| PKG1 | Cell Signaling | 3248S | 1:1000 |
| PKG1a | Cell Signaling | 13511 | 1:1000 |
| TITIN | Affinity Biosciences | DF12549 | 1:2000 |
| TITIN | Abcam | Ab284860 | 1:1000 |
| Polyclonal Goat Anti-Rabbit Immunoglobulins HRP | Agilent technologies | P0448 | 1:5000 |
| Polyclonal Rabbit Anti-Goat Immunoglobulins HRP | Agilent technologies | P0449 | 1:5000 |
